# Exploring Molecular Alterations in Breast Cancer Among Indian Women Using Label-Free Quantitative Serum Proteomics

**DOI:** 10.1155/bri/5584607

**Published:** 2024-11-28

**Authors:** Anil Kumar Tomar, Ayushi Thapliyal, Sandeep R. Mathur, Rajinder Parshad, Suhani, Savita Yadav

**Affiliations:** ^1^Department of Biophysics, All India Institute of Medical Sciences, New Delhi 110029, India; ^2^Department of Pathology, All India Institute of Medical Sciences, New Delhi 110029, India; ^3^Department of Surgical Disciplines, All India Institute of Medical Sciences, New Delhi 110029, India

## Abstract

The clinical data indicate that diverse parameters characterize breast cancer patients in India, including age at presentation, risk factors, outcomes, and behavior. Alarming incidence and mortality rates emphasize the crucial need for early screening measures to combat breast cancer-related deaths effectively. Quantitative proteomic approaches prove pivotal in predicting cancer prognosis, analyzing protein expression patterns tied to disease aggressiveness and metastatic potential, and facilitating conversant therapy selection. Thus, this study was envisioned with the goal of identifying protein markers associated with breast cancer in Indian women, which could potentially be developed as diagnostic tools and therapeutic targets in the future. Applying label-free proteomic quantitation method and statistical analysis, several differentially expressed proteins (DEPs) were identified in the serum of breast cancer patients compared to controls, including SBSN, ANG, PCOLCE, and WFDC3 (upregulated), and PFN1, FLNA, and DSG2 (downregulated). The expression of SBSN was also validated by western blotting. Statistical methods were employed to proteomic expression data, which highlighted the ability of DEPs to distinguish between breast cancer and control samples. Conclusively, this study recognizes prospective biomarkers for breast cancer among Indian women and highlights the requisite of in-depth functional studies to elucidate their precise roles in breast cancer development. We particularly emphasize on SBSN and PFN1, as these proteins were observed to be progressively overexpressed and under expressed, respectively, in breast cancer samples compared to control samples, ranging from early-stage to metastatic cases.

## 1. Introduction

According to GLOBOCAN 2020 report, breast cancer in women is the most commonly diagnosed cancer that accounts for 11.7% of all cancer cases globally. In 2020, more than 2 million new cases of breast cancer were diagnosed, and the disease led to 685,000 deaths [1]. In Indian population too, it is the most prevalent malignancy among women [2]. Between 2008 and 2012, India had rampant incidences of breast cancer, with an 11.54% increase in incidence and a 13.82% increase in deaths [3, 4]. According to existing literature, a majority of breast cancer patients in India differ in parameters, such as age at presentation, risk factors, outcome, and behavior from those described in western nations [5, 6]. The traditional reproductive risk factors associated with the illness in western population, such as early menarche, late menopause, nulliparity, and insufficient breastfeeding, are mostly absent in them.

Breast cancer is a heterogeneous disease with no organ of origin-specific treatment or outcome predictors. The currently used clinical molecular classification of breast cancer based on the receptor profiling serves as a surrogate marker to help guide treatment and predict prognosis [7–9]. The quest for improved breast cancer outcomes necessitates ongoing efforts to uncover and validate novel biomarkers, particularly those conducive to early detection. There have been advances in the understanding mRNA transcripts over the last two decades with the introduction of set of 50 transcripts, commonly referred to as the PAM50 transcript. However, comparisons between transcript levels and corresponding protein expression have revealed that mRNA and protein levels may not always show a strong correlation [10]. Given that most therapeutic strategies focus on targeting and modifying protein function and activity, it is crucial for proteomics to play a central role in understanding the mechanisms driving tumorigenesis in breast cancer [11, 12].

Recent studies have shown that in breast cancer compared to normal samples, there is differential expression of proteins involved in key molecular pathways such as angiogenesis, cell cycle regulation, and metastatic mechanisms [13–15]. These differentially expressed proteins (DEPs) may also act as biomarkers for disease, and their pathways act as potential therapeutic targets. However, most of the advances on proteomics and gene transcripts come from western literature. Indian breast cancer patients are phenotypically different from the west with younger age at presentation, higher grade tumors, and higher proportion of triple negative tumors [16, 17]. They additionally lack the classical reproductive risk factors described in the English literature [5, 18]. Keeping the differences in disease presentation, we conducted this study to identify the differentially expressed serum proteins in breast cancer patients. The ultimate goal was to identify potential markers for the classification of patients exhibiting variable disease behavior, which could then be investigated further for more aggressive multimodality disease management. Furthermore, elucidation of the mechanisms linked to DEPs in these women can aid in the identification of potential therapeutic targets.

## 2. Materials and Methods

### 2.1. Ethical Permission and Sample Collection

**Figure 1:**
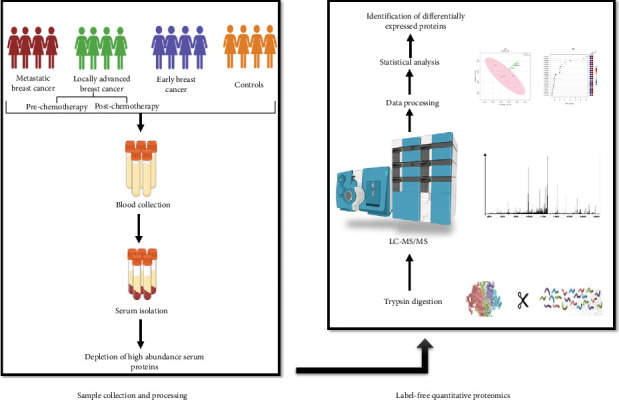
Study design.

The study participants were recruited following ethical approval from the Institutional Ethics Committee, All India Institute of Medical Sciences, New Delhi (File No. IEC-645/03.07.2020). Blood samples were collected from individuals diagnosed with early breast cancer (EBC), locally advanced breast cancer (LABC), metastatic breast cancer (M), as well as from healthy volunteer controls (C), after obtaining their signed informed consent forms. Their age was between 38 and 73 years. In LABC group, samples were collected two times-prechemotherapy (LAB_V1), and postchemotherapy (LAB_V2).  Table 1 provides additional details about breast cancer patients. In total, 21 samples (C = 5, EBC = 5, LAB_V1 = 4, LAB_V2 = 4, and M = 3) were processed for proteomics analysis.  Figure 1 provides an overview of the experimental design.

The cases were evaluated by detailed history, clinical examination, bilateral mammogram, core needle biopsy of breast lesion, core needle biopsy/FNAC of axillary node as applicable, and staging investigations done as per the existing standard protocol practices. For this study, EBC included tumors up to and including 5 cm in maximum dimension with or without single mobile axillary lymph node involvement. Tumors more than 5 cm in maximum dimension with or without axillary lymph node involvement and/or skin changes were classified as LABC. Prechemotherapy blood samples were collected before the start of any systemic treatment, and postchemotherapy blood samples were collected 2-3 weeks after completion of third cycle neoadjuvant chemotherapy (NACT).

### 2.2. Label-Free Quantitative Proteomics

#### 2.2.1. Sample Processing, Depletion of Top Abundant Proteins, and Trypsin Digestion

Blood samples were drawn into Vacutainers and left at room temperature for 30 min to facilitate clot formation. Samples were then centrifuged at 1000*g* for 10 min at 4°C to remove the clot from the serum [19]. Serum (supernatant) was collected in fresh tubes. Analysis of human serum and other biological fluids is often complicated by the presence of high concentrations of various proteins, including albumin and IgG. These high abundance proteins make up more than 70% of total serum proteins. To effectively study the expression of most of the proteins, high-abundance proteins were depleted from the serum samples using commercially available depletion columns (High Select Depletion Spin Columns, Thermo Scientific). After depletion, 50 *μ*g of serum sample was processed for digestion. First, the samples were treated with 5 mM TCEP for reduction and 50 mM iodoacetamide for alkylation, followed by incubation with trypsin for overnight digestion at 37°C. The digested peptides were then purified by C18 columns and vacuum dried.

#### 2.2.2. Mass Spectrometric Analysis and Data Processing

Mass spectrometry analyses were conducted using an Easy-nLC-1000 system in tandem with an Orbitrap Exploris mass spectrometer (Thermo Fisher Scientific), following the methodology outlined in previous studies [20, 21]. Briefly, 1  *μ*g of peptides were separated on a C18 column (15 cm, 3.0 *μ*m Acclaim PepMap) at a flow rate of 500 nL/min for 60 min, followed by acquisition of MS1 and MS2 spectra. The raw spectra files obtained were then processed for protein identification by searching against the UniProt human database and abundance estimation using Proteome Discoverer (v2.4).

### 2.3. Statistical Analysis

To identify significant proteomic alterations, statistical analysis was conducted using the methods outlined in our prior studies [22, 23]. The statistical methods, including volcano plot analysis, principal component analysis (PCA), hierarchical clustering analysis (HCA), partial least squares-discriminant analysis (PLS-DA), and orthogonal projections to latent structures discriminant analysis (OPLS-DA), were employed using MetaboAnalyst 5.0 [24]. After adding missing values using KNN imputation method, data were normalized by Pareto scaling prior to analysis. PCA and HCA are unsupervised methods, while PLS-DA and OPLS-DA are supervised methods. PCA identifies the principal axes responsible for the variance in the data, making it particularly useful for detecting outliers. The variance in the data is represented through scores, which are plotted to highlight differences between groups. In contrast, HCA groups data points with similar characteristics into clusters. PLS-DA applies multivariate regression analysis to identify key features (such as DEPs) by calculating a variable importance in projection (VIP) score. Proteins with a VIP score ≥ 1 are considered DEPs. OPLS-DA is a robust technique that simultaneously reduces dimensionality and identifies DEPs driving the separation between groups. Compared to PLS-DA, OPLS-DA offers enhanced capability in distinguishing between variations in the dataset that are most relevant for predicting group differences.

### 2.4. Western Blotting

Differential expression of suprabasin (SBSN) protein was validated in different samples by western blotting. To do this, 25 µg proteins were first separated on a 12% SDS-PAGE gel and then transferred to a PVDF membrane (100 V, 1 h). Following transfer, blot was blocked with nonfatty milk and then incubated in primary antibodies (antihuman SBSN, MyBioSource) for overnight at 4°C. Next day, blot was incubated in secondary antibodies for 2 h at RT, followed by a thorough washing. The protein bands were detected by ECL solution, and images were acquired on gel documentation system (Syngene, Cambridge, UK).

## 3. Results

As outlined in the methodology section, 21 samples were processed by tandem mass spectrometry for identification and differential quantitation of proteins, identifying a total of 6103 peptide groups corresponding to 618 proteins (Supporting file 1). Proteins quantified in less than 50% of the samples within each group were not included in the differential analysis. Abundance data were then processed to add missing values by the KNN method to ensure completeness of the dataset. To identify statistically significant DEPs, volcano plots were constructed by plotting the log2 fold change (FC) values and the negative logarithm of the *p*-values on the *X* and *Y* axes, respectively, with thresholds for FC and *p*-value being set at 2 and 0.05, respectively. Proteins with a higher relative abundance in a specific group were classified as upregulated or overexpressed, and those with a lower relative abundance as downregulated or under expressed.

### 3.1. Breast Cancer vs. Controls

**Figure 2:**
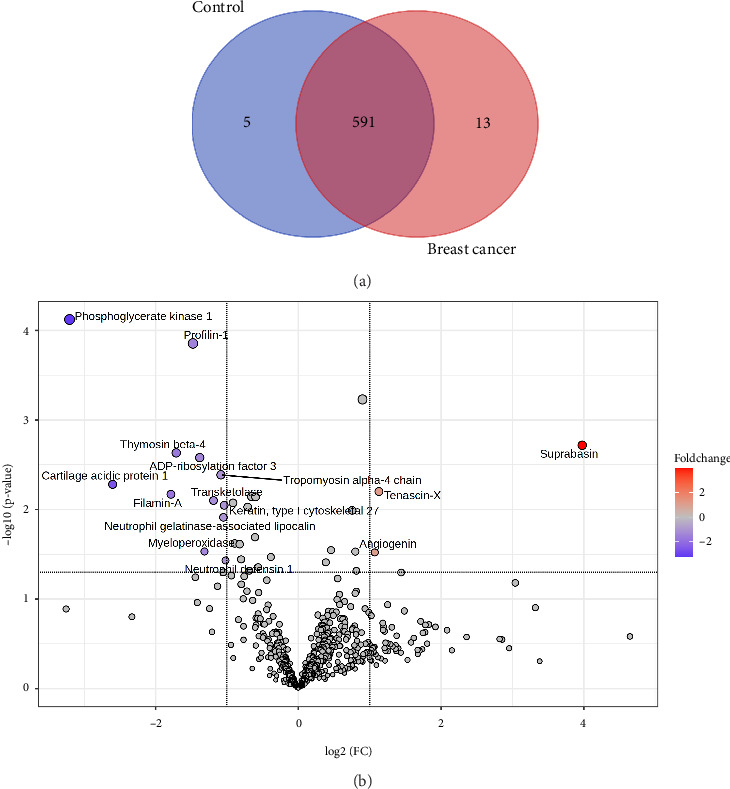
Breast cancer vs. controls. (a) Venn diagram showing protein distribution; and (b) differentially expressed proteins (DEPs) identified by volcano plot.

Initially, differential proteomic analysis was performed between control (*n* = 5) and breast cancer (*n* = 12, consisting of 5 EBC, 4 LABC, and 3 metastasis) groups. In total, 609 proteins were quantified in these two groups, with 591 found in both, and 13 and 5 proteins exclusively detected in breast cancer and control samples, respectively (Figure 2(a)). Of the 15 DEPs identified, 3 were upregulated, and 12 were downregulated in breast cancer samples compared to controls (Figure 2(b)). In breast cancer samples, SBSN, tenascin, and angiogenin (ANG) were found upregulated, while phosphoglycerate kinase, filamin-A (FLNA), thymosin beta-4, profilin-1 (PFN1), ADP-ribosylation factor 3, myeloperoxidase, neutrophil gelatinase-associated lipocalin, and neutrophil defensin 1 exhibited downregulated expression (Table 2).

**Figure 3:**
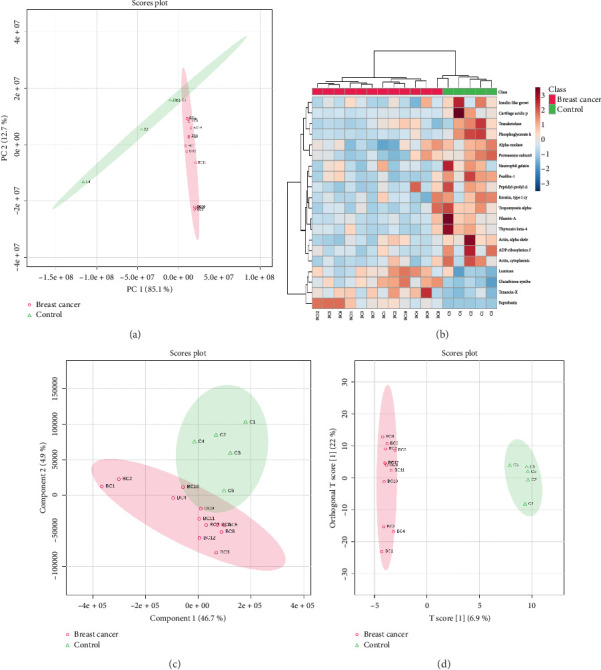
Breast cancer vs. controls: Statistical analysis of differentially expressed proteins. (a) Principal component analysis considering only significant DEPs identified by volcano plot analysis; (b) hierarchical clustering analysis using top 20 classifiers; (c) partial least squares-discriminant analysis (PLS-DA) scores plot; and (d) orthogonal projections to latent structures discriminant analysis (OPLS-DA) scores plot.

When all identified proteins were considered, no clear separation between the two groups was observed in the PCA score plot (Figure S1a). However, the PCA score plot based on only the DEPs successfully separated the breast cancer and control groups (Figure 3(a)). HCA based on expression of all the quantified proteins produced mixed groups, when only the top DEPs were utilized for clustering, the samples correctly grouped into distinct clusters of breast cancer and controls (Figure 3(b)). A clear separation was observed between the breast cancer and control cohorts in the PLS-DA score plot, underscoring the statistical potential of identified DEPs in distinguishing these two groups (Figure 3(c)). A total of 67 proteins exhibited a VIP score of 1 or more. Albumin, apolipoprotein, complement C3 and C4-B, alpha-1-antitrypsin, immunoglobulins, bleomycin hydrolase, alpha-2-macroglobulin, trypsin-1, haptoglobin, inter-alpha-trypsin inhibitor, guanylate cyclase soluble subunit alpha-2, myosin-3, cathepsin G, and vitamin D-binding protein were listed as the top classifiers (Figure S1b). Similarly, a distinct separation was observed between these two groups in the OPLS-DA score plot, which further validates the classification power of the identified DEPs (Figure 3(d)). A total of 199 proteins were sorted with a VIP score of 1 or greater, with phosphoglycerate kinase 1 (PGK1), PFN1, glutathione synthetase, thymosin beta-4, ADP-ribosylation factor 3, actin, SBSN, tropomyosin alpha-4 chain, transketolase, neutrophil gelatinase-associated lipocalin, FLNA, cartilage acidic protein 1, proteasome subunit beta type-6, lumican, tenascin-X, and myeloperoxidase emerging as the top classifiers (Figure S1c).

#### 3.1.1. EBC vs. Controls

Collectively, 613 proteins were quantified in these two groups, of which 571 were present in the both groups, while 14 and 28 proteins were exclusively measured in EBC and control samples, respectively (Figure S2a). A total of 15 proteins were found differentially expressed, with 8 proteins showing upregulation and 7 proteins exhibiting downregulation in EBC samples compared to controls (Figure S2b). SBSN, immunoglobulin heavy variable 3–49 and 3–64, purine nucleoside phosphorylase, procollagen C-endopeptidase enhancer 1 (PCOLCE), calmodulin-like proteins 3 and 5, and nidogen-1 were found upregulated, while PGK1, FLNA, thymosin beta-4, PFN1, ADP-ribosylation factor 3, tropomyosin alpha-4 chain, and macrophage receptor MARCO were downregulated in EBC samples (Supporting file 2).

When all identified proteins were considered, the PCA score plot displayed no clear separation between the two groups (Figure S2c). However, the PCA score plot based on DEPs was able to effectively classify the two groups (Figure S2d). HCA based on expression of all the quantified proteins produced mixed groups, while analysis based on top DEPs effectively distinguished EBC samples from control samples, grouping them into separate clusters (Figure S2e). A clear separation was observed between EBC and control cohorts in the PLS-DA scores plot, validating the classification power of the protein expression data (Figure S2f). A total of 36 proteins exhibited a VIP score of 1 or greater. Albumin, alpha-2-macroglobulin, serotransferrin, apolipoprotein A-I, haptoglobin, vitamin D-binding protein, immunoglobulin heavy constants (gamma 1, mu, alpha 1, kappa, gamma 3, and lambda 2), bleomycin hydrolase, and trypsin-1 were found among the top classifiers (Figure S2g). The OPLS-DA score plot also clearly distinguished between EBC and control samples (Figure S2h). The number of proteins identified with a VIP score of 1 or greater was 217. Thymosin beta-4, tropomyosin alpha-4 chain, PGK1, SBSN, PCOLCE, purine nucleoside phosphorylase, immunoglobulin heavy variable 3–64, calmodulin-like protein 3, lumican, plasma serine protease inhibitor, alpha-enolase, heat shock cognate 71 kDa protein, nucleophosmin, and glutathione synthetase were the top classifiers (Figure S2i).

#### 3.1.2. LABC (LAB_V1) vs. Controls

Collectively, 609 proteins were quantified in these two groups, of which 577 were present in the both groups, while 10 and 22 proteins were exclusively measured in LABC and control samples, respectively (Figure S3a). Overall, 16 proteins were identified as differentially expressed, with 7 proteins showing upregulation and 9 proteins showing downregulation in LABC samples compared to controls (Figure S3b). SBSN, bleomycin hydrolase, immunoglobulin lambda variable 8–61, ANG, tenascin-X, fetuin-B, and periostin were found upregulated, while PGK1, immunoglobulin heavy variables 4–34 and 3–64D, transketolase, desmoglein-2 (DSG2), actin, alpha skeletal muscle, ADP-ribosylation factor 3, protein S100-A6, and PFN1 were downregulated in EBC samples (Supporting File 2).

PCA scores plot considering all the identified proteins displayed no separation between these groups (Figure S3c). However, PCA score plot based on DEPs was able to effectively classify the two groups (Figure S3d). HCA based on expression of top proteins clustered samples into EBC and control groups (Figure S3e). The PLS-DA and OPLS-DA score plots both demonstrated a distinct separation between LABC and control samples (Figures S3f and S3h). A total of 69 proteins exhibited PLS-Da VIP score of 1 or greater, and bleomycin hydrolase, trypsin-1, albumin, apolipoprotein A-I, ceruloplasmin, fibronectin, hemopexin, C4b-binding protein alpha chain, alpha-1B-glycoprotein, transthyretin, complement component C9, and apolipoprotein A-II were the top classifiers (Figure S3g). In case of OPLS-DA, the number of proteins with VIP score ≥ 1 was 191 and top classifiers were apolipoprotein A-II, PFN1, PGK1, immunoglobulin heavy variable 4–34, DSG2, protein S100-A6, SBSN, fetuin-B, ANG, periostin, glutathione synthetase, von Willebrand factor, tropomyosin alpha-3 chain, receptor-type tyrosine-protein phosphatase gamma, and guanylate cyclase soluble subunit alpha-2 (Figure S3i).

#### 3.1.3. Metastasis vs. Controls

Collectively, 607 proteins were quantified in these two groups, of which 566 were present in the both groups, while 8 and 33 proteins were exclusively measured in M and control samples, respectively (Figure S4a). A total of 23 proteins were found to be differentially expressed in M samples compared to controls, with 9 showing upregulation and 14 downregulation (Figure S4b). SBSN, immunoglobulin lambda variable 8–61, bleomycin hydrolase, HLA class I histocompatibility antigen, A alpha chain, immunoglobulin heavy constant gamma 4, Ras-related protein Rab-10, WAP four-disulfide core domain protein 3 (WFDC3), Golgi membrane protein 1, and tenascin-X were found upregulated, while PGK1, keratin, PFN1, transaldolase, F-box only protein 50, protein-glutamine gamma-glutamyltransferase K, proteasome subunit alpha type-3, ADP-ribosylation factor 3, protein S100-A14, immunoglobulin heavy variable 4–34, tropomyosin alpha-4 chain, histidine ammonia-lyase, and di-N-acetylchitobiase were downregulated in M samples (Supporting File 2).

PCA scores plot considering all the identified proteins displayed no separation between these groups (Figure S4c). However, the PCA score plot based on DEPs was able to effectively classify the two groups (Figure S4d). HCA based on expression of top DEPs clustered samples into M and control groups correctly (Figure S4e). A distinct separation was observed between M and control samples in the PLS-DA score plot (Figure S4f). A total of 52 proteins exhibited a VIP score of 1 or higher and the list of top classifiers included apolipoprotein B-100, complement C3, complement C4-B, albumin, keratin, haptoglobin, alpha-1-antitrypsin, apolipoprotein A-I, hemopexin, kininogen-1, transthyretin, alpha-2-HS-glycoprotein, immunoglobulin heavy constant alpha 1, alpha-1-acid glycoprotein 1, and bleomycin hydrolase (Figure S4g). Notably, this group includes several serum proteins that are typically found in high abundance. In breast cancer patients, most of these proteins show even greater levels compared to the control group, including immunoglobulin heavy constant alpha 1 (FC = 1.69), albumin (FC = 1.51), haptoglobin (FC = 2.08), alpha-1-acid glycoprotein 1 (FC = 3.50), and bleomycin hydrolase (FC = 6.59). The OPLS-DA scores plot also successfully distinguished between M and control cohorts (Figure S4h). A total of 202 proteins were sorted with a VIP score of 1 or greater. The list of the top classifiers included PFN1, histidine ammonia-lyase, keratin, bleomycin hydrolase, immunoglobulin heavy variable 4–34, protein-glutamine gamma-glutamyltransferase K, F-box only protein 50, von Willebrand factor, immunoglobulin heavy constant gamma 4, leucine-rich alpha-2-glycoprotein, plasma serine protease inhibitor, tenascin-X, SBSN, HLA class I histocompatibility antigen, and WFDC3 (Figure S4i).

### 3.2. LABC (LAB_V1) vs. EBC

**Figure 4:**
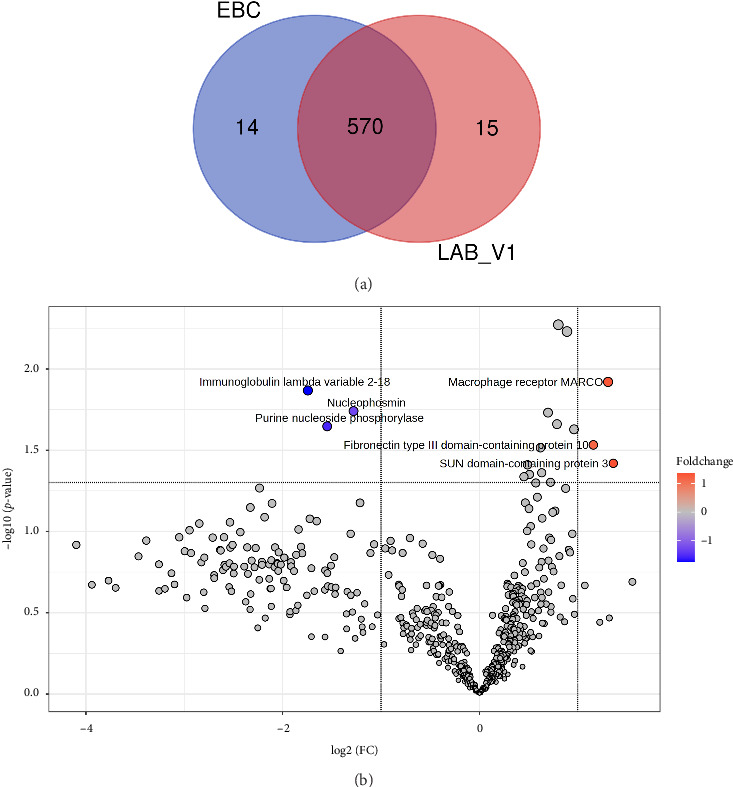
Prechemotherapy locally advanced breast cancer (LAB_V1) vs. early breast cancer (EBC). (a) Venn diagram showing protein distribution; (b) differentially expressed proteins (DEPs) identified by volcano plot.

In total, 599 proteins were quantified in these two groups, of which 570 were present in the both groups, while 14 and 15 proteins were exclusively quantified in EBC and LAB_V1 samples, respectively (Figure 4(a)). Only 6 DEPs were identified, with 3 proteins showing upregulation and 3 proteins showing downregulation (Figure 4(b), Supporting file 2).

**Figure 5:**
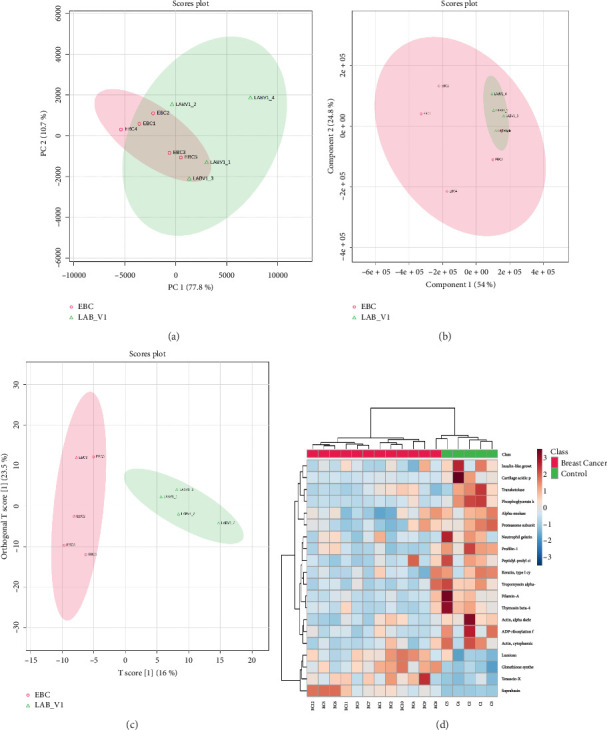
Prechemotherapy locally advanced breast cancer (LAB_V1) vs. early breast cancer (EBC): Statistical analysis of differentially expressed proteins. (a) Principal component analysis considering only significant DEPs identified by volcano plot analysis; (b) partial least squares-discriminant analysis (PLS-DA) scores plot; (c) orthogonal projections to latent structures discriminant analysis (OPLS-DA) scores plot; (d) hierarchical clustering analysis using top 20 classifiers.

PCA and PLS-DA score plots failed to separate these groups (Figures 5(a) and 5(b)). However, OPLS-DA clearly separated LAB_V1 and EBC groups (Figure 5(c)). Further, OPLS-DA identified 238 proteins with a VIP score of 1 or greater, and C4b-binding protein, thyroxine-binding globulin, SUN domain-containing protein 3, nucleophosmin, fibronectin type III domain-containing protein 10, coagulation factor V, apolipoprotein A-II, complement C5, corticosteroid-binding globulin, platelet-activating factor acetylhydrolase, and collectin-10 were listed as the top classifiers (Figure S5). Also, HCA based on expression of all the quantified proteins clustered samples into EBC and LAB_V1 groups correctly (Figure 5(d)).

### 3.3. Metastasis vs. LABC (LAB_V1)

**Figure 6:**
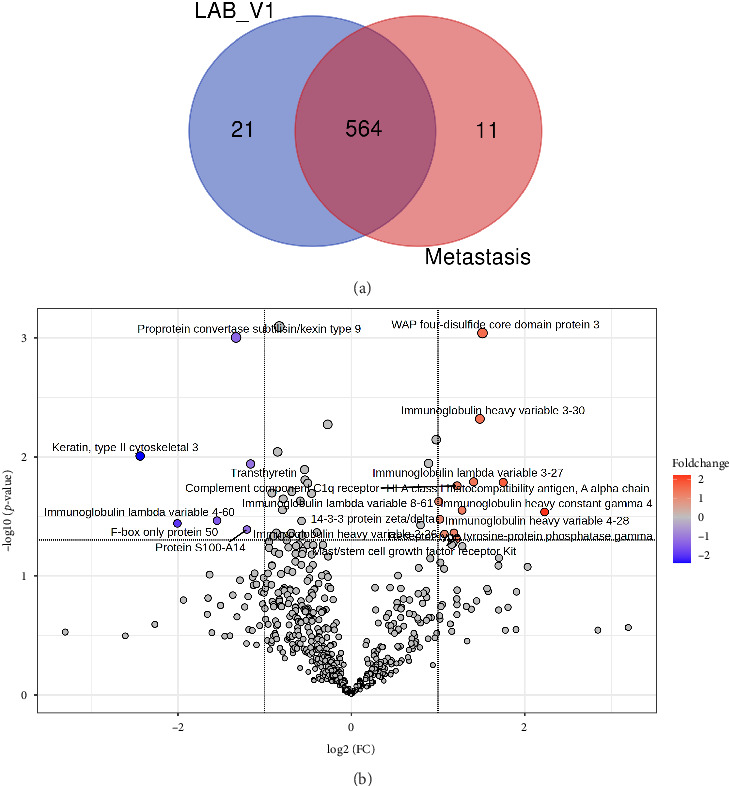
M vs. prechemotherapy locally advanced breast cancer (LAB_V1). (a) Venn diagram showing protein distribution; (b) differentially expressed proteins (DEPs) identified by volcano plot.

In total, 596 proteins were quantified in these two groups, of which 564 were present in the both groups, while 11 and 21 proteins were exclusively quantified in metastasis and LAB_V1 samples, respectively (Figure 6(a)). A total of 18 proteins were found differentially expressed, with 12 proteins being upregulated and 6 proteins being downregulated in metastasis samples (Figure 6(b)).

**Figure 7:**
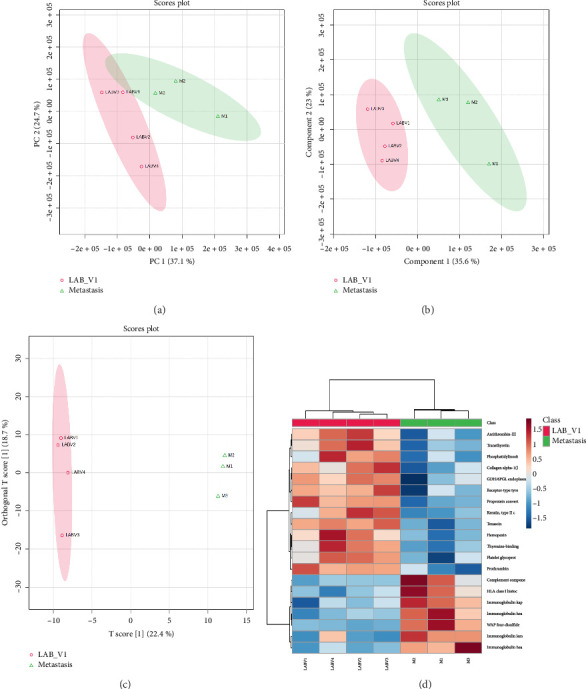
M vs. prechemotherapy locally advanced breast cancer (LAB_V1): Statistical analysis of differentially expressed proteins. (a) Principal component analysis considering only significant DEPs identified by volcano plot analysis; (b) partial least squares-discriminant analysis (PLS-DA) scores plot; (c) orthogonal projections to latent structures discriminant analysis (OPLS-DA) scores plot; (d) hierarchical clustering analysis using top 20 classifiers.

The upregulated proteins included immunoglobulins, HLA class I histocompatibility antigen, WFDC3, complement component C1q receptor, mast/stem cell growth factor receptor kit, receptor-type tyrosine-protein phosphatase gamma, and 14-3-3 protein zeta/delta. Conversely, F-box only protein 50, proprotein convertase subtilisin/kexin type 9, protein S100-A14, and transthyretin were downregulated (Supporting file 2). The score plots generated by PCA, PLS-DA, and OPLS-DA all revealed a clear separation between LABC and metastasis groups (Figures 7(a), 7(b), 7(c)). Also, HCA correctly clustered samples into LABC and M groups (Figure 7(d)). PLS-DA and OPLS-DA identified 48 and 234 proteins with VIP score ≥ 1, respectively.

The top classifiers identified by PLS-DA included albumin, complement C3, hemopexin, apolipoprotein B-100, transthyretin, haptoglobin, alpha-2-macroglobulin, kininogen-1, ceruloplasmin, and clusterin (Figure S6a). The top classifiers based on VIP score in OPLS-DA included proprotein convertase subtilisin/kexin type 9, WFDC3, tenascin, prothrombin, immunoglobulins, thyroxine-binding globulin, transthyretin, complement component C1q receptor, hemopexin, HLA class I histocompatibility antigen, collagen alpha-1(III) chain, and clusterin (Figure S6b).

### 3.4. LABC, Pre- vs. Postchemotherapy

**Figure 8:**
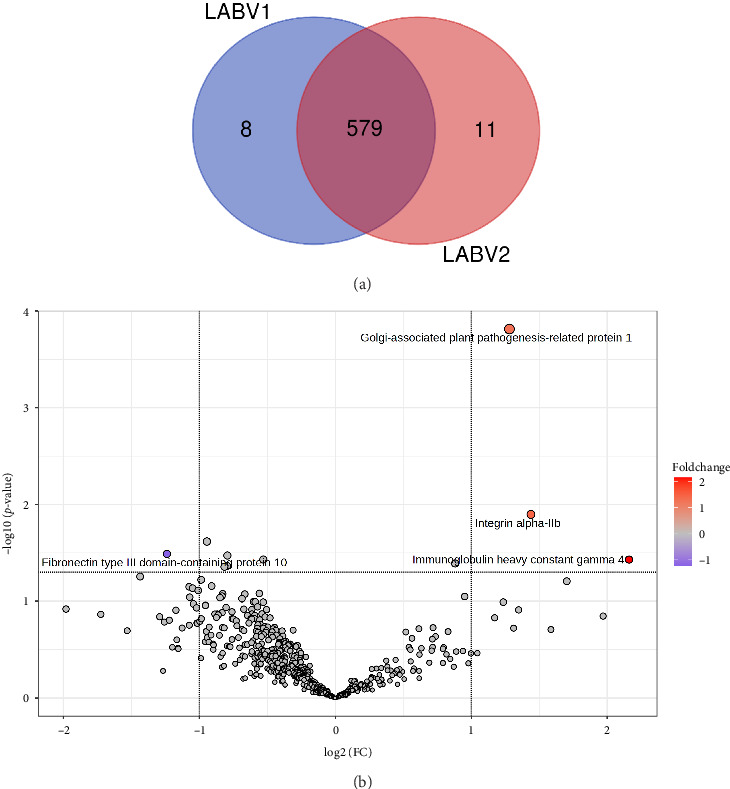
Postchemotherapy locally advanced breast cancer (LABV2) vs. prechemotherapy locally advanced breast cancer (LABV1). (a) Venn diagram showing protein distribution; (b) differentially expressed proteins (DEPs) identified by volcano plot.

**Figure 9:**
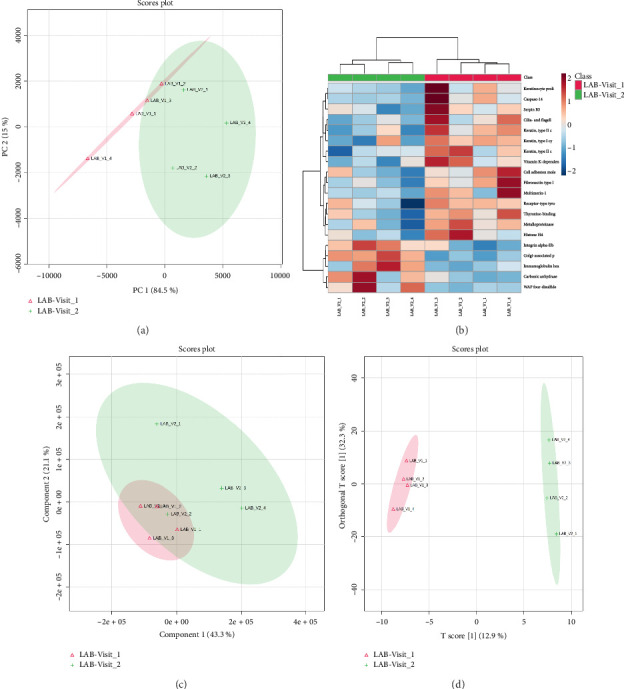
Postchemotherapy locally advanced breast cancer (LABV2) vs. prechemotherapy locally advanced breast cancer (LABV1): Statistical analysis of differentially expressed proteins. (a) Principal component analysis considering only significant DEPs identified by volcano plot analysis; (b) hierarchical clustering analysis using top 20 classifiers; (c) partial least squares-discriminant analysis (PLS-DA) scores plot; (d) orthogonal projections to latent structures discriminant analysis (OPLS-DA) scores plot.

Collectively, 598 proteins were quantified in these two groups, of which 579 were present in the both groups, while 8 and 11 proteins were exclusively quantified in pre- and postchemotherapy LABC samples, respectively (Figure 8(a)). Out of the 4 DEPs identified in postchemotherapy LABC samples, 3 proteins were upregulated, while 1 protein was downregulated compared to the prechemotherapy LABC samples (Figure 8(b)). Immunoglobulin heavy constant gamma 4, integrin alpha-IIb, and Golgi-associated plant pathogenesis-related protein 1 were found upregulated, and fibronectin type III domain-containing protein 10 was downregulated in postchemotherapy LABC samples (Supporting file 2). PCA scores plot considering all the identified proteins displayed no separation between these groups; however, the PCA score plot utilizing DEPs classified the two groups distinctly (Figure 9(a)). HCA based on expression of all the quantified proteins produced mixed groups. However, when only the top DEPs were utilized for clustering, the samples correctly grouped into distinct clusters of pre- and postchemotherapy LABC (Figure 9(b)). The PLS-DA score plot did not fully distinguish between pre- and postchemotherapy LABC samples (Figure 9(c)). A total of 54 proteins were computed with a VIP score of 1 or greater. The top classifiers were fibronectin, complement C3, ceruloplasmin, keratin, apolipoprotein A, haptoglobin, antithrombin-III, vitamin D-binding protein, hemopexin, inter-alpha-trypsin inhibitor heavy chain H1, kininogen-1, plasma protease C1 inhibitor, transthyretin, immunoglobulin heavy constant mu, and heparin cofactor 2 (Figure S7a). In contrast to the PLS-DA, the OPLS-DA score plot effectively differentiated between pre- and postchemotherapy LABC samples (Figure 9(d)). A total of 217 proteins were computed with a VIP score of 1 or greater, and thyroxine-binding globulin, immunoglobulin heavy constant gamma 4, integrin alpha-IIb, fibronectin type III domain-containing protein 10, keratin, caspase-14, vitamin K-dependent protein Z, multimerin-1, histone H4, carbonic anhydrase 2, metalloproteinase inhibitor 1, transketolase, cathepsin B, and cell adhesion molecule 1 were the top classifiers (Figure S7b).

#### 3.4.1. Western Blotting

**Figure 10:**
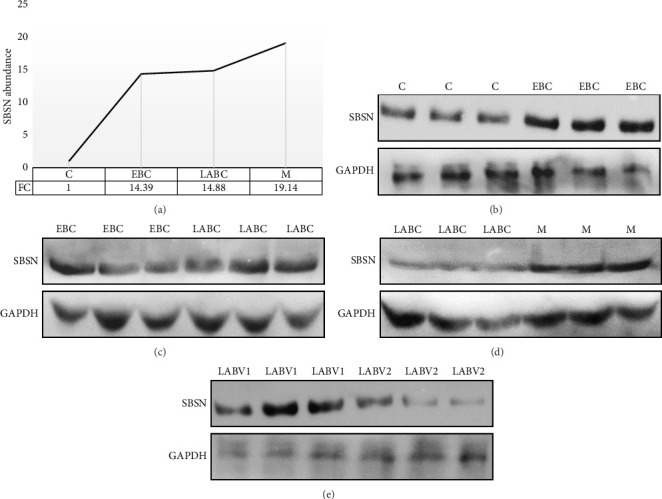
(a) Relative fold change in SBSN abundance in early breast cancer (EBC), locally advanced breast cancer (LABC), and metastatic breast cancer (M) compared to controls (C) as examined by quantitative proteomics; (b–e) Western blot analysis of suprabasin (SBSN) expression in breast cancer samples: (b) EBC vs C; (c) LABC vs EBC; (d) M vs LABC; and (e) postchemotherapy locally advanced breast cancer (LABV2) vs. prechemotherapy locally advanced breast cancer (LABV1).

The varying expression of SBSN protein, as identified in breast cancer samples by label-free proteome analysis, was confirmed by western blot analysis (Figures 10(a), 10(b), 10(c), 10(d), 10(e); Supporting file S3). As depicted by the intensity of bands on blot, SBSN expression showed a progressive increase in breast cancer samples compared to controls, with levels rising from early stage to locally advanced, and further to metastatic cases. Similar to the proteomics data, the difference between EBC and LABC samples was relatively very low compared to the other groups.

## 4. Discussion

Proteomics has become a cutting-edge area of study, providing a thorough and comprehensive knowledge of the molecular complexities underlying the complex diseases by deciphering the diverse and dynamic protein profiles of a biological system [25, 26]. The breast cancer proteomics can contribute toward prognosis, diagnosis, and the identification of new therapeutic targets. Early diagnosis is critical for improving patient outcomes, and proteomic analyses of breast cancer tissues and proteomics have led to the discovery of novel and promising biomarkers for early detection. For instance, the overexpression of human epidermal growth factor receptor 2 (HER2), progesterone receptor (PR), and estrogen receptor (ER) has been identified as crucial biomarkers guiding the diagnosis and classification of breast cancer subtypes [8, 27]. These biomarkers not only aid in early detection but also contribute to the development of targeted therapies tailored to specific molecular subtypes. Quantitative proteomic approaches play a pivotal role in predicting the prognosis of cancer patients via analysis of protein expression patterns linked to disease aggressiveness and propensity for metastasis, which, in turn, can assist in therapy selection. Various proteins associated with DNA repair, angiogenesis, and cell cycle control have been identified as prognostic indicators that provide information about the probable course of the illness [28]. This information enables clinicians to stratify patients based on their individual risk profiles, facilitating more personalized and effective treatment strategies.

Here, we performed label free quantitative proteomic analysis to study the impact of advancing breast cancer stages on serum proteome. In total, 15, 16, and 23 DEPs were identified in early stage, locally advanced, and M samples, respectively, compared to healthy controls. Stringent statistical analysis was performed to select significant DEPs with a potential to serve as general or stage-specific biomarkers of breast cancer. For example, SBSN expression was found upregulated in breast cancer samples compared to controls, with maximum expression in metastatic cases. Similarly, PFN1 expression showed a consistent reduction in cancer samples with minimum expression in metastatic cases. On the other hand, PCOLCE, ANG, and WFDC3 were precisely upregulated in early stage, locally advanced, and M samples, respectively. The statistical power of the identified DEPs to classify breast cancer sample groups was underlined by a number of methods such as PCA, hierarchical clustering, PLS-DA, and OPLS-DA. Bioinformatics analysis of DEPs in breast cancer patients revealed their involvement in various pathways, which are closely associated with cancer progression. These include platelet degranulation, response to elevated platelet cytosolic Ca^2+^, platelet activation, signaling and aggregation, neutrophil degranulation (as shown by reactome pathways). Additionally, two of the downregulated proteins, myeloperoxidase and neutrophil defensin, are linked to transcriptional misregulation in cancer (according to KEGG pathways).

One of the upregulated proteins identified in breast cancer samples, SBSN is a secreted protein involved in a complex array of cellular functions, including but not limited to invasion, migration, angiogenesis, apoptosis, immune evasion, proliferation, and metastasis. It plays a key part in these biological processes and influences the dynamic equilibrium of cellular responses [29]. The conclusions drawn from various expression studies in cancer patient cohorts suggest that SBSN is an oncogene that plays a crucial role in the development and progression of several cancer types [29–35]. More specifically, increased SBSN expression was linked to advanced disease development, a shorter survival time, and poor prognosis. Zhu et al. reported that SBSN was overexpressed in esophageal squamous cell carcinoma and linked to proliferation and tumorigenicity [35]. Similarly, Houri et al. reported that SBSN increases the invasion and migration capabilities of oral squamous cell carcinoma cells [29]. Another recent study, utilizing 2D gel-based proteomics analysis, identified SBSN as a novel and potential candidate biomarker of endometrial cancer [36]. While there are several reports correlating SBSN overexpression with development and progression in different cancer types, such reports pertaining to breast cancer are limited.

In present study, proteomics data showed a higher expression of SBSN in breast cancer samples compared to controls; however, significant difference was not observed between breast cancer groups, EBC to LABC or LABC to metastasis. This can be attributed to small sample size, a limitation of this study. Further, we validated SBSN expression orthogonally using western blotting, which confirmed SBSN over expression in breast cancer samples compared to controls. Moreover, serum samples from EBC to LABC to M cases showed a sequentially higher abundance of SBSN. Recent research shows that SBSN plays a significant role in various processes related to tumor behavior, such as cell proliferation, migration, angiogenesis, immune evasion, and metastasis, and its inhibition may lead to significant reduction in proliferation, invasion, and metastasis of cancer cells, implying its potential role in cancer progression [35, 37, 38]. The sequential overexpression of SBSN, as observed in this study, suggests its involvement in breast cancer progression possibly by modulating signaling pathways that promote proliferation of cancer cells, invasion, and metastasis. These findings conclude that SBSN may act as an oncogenic factor linked to the progression of breast cancer and warrants further investigations to explore it as a potential biomarker.

PFN1, in contrast to SBSN, exhibited a decreasing expression in serum samples of breast cancer from EBC to metastasis. This finding is consistent with other prior research that demonstrated a correlation between the advancement of breast cancer and the loss or downregulation of PFN1 expression [39, 40]. PFN1 is an actin-binding protein made up of 135 amino acids and expressed ubiquitously in almost all cell and tissue types [41]. Interestingly, actin was also found downregulated in LABC samples (Table). PFN1 is involved in various cancer-associated cellular processes, such as tumor proliferation, immune response, apoptosis, and metastasis [42–44]. In addition to breast cancer, downregulated expression of PFN1 has also been reported in other cancers including non-small cell lung cancer, renal cell cancer, pancreatic cancer, and hepatocellular carcinoma [42, 45–48]. According to these studies, PFN1 downregulation promotes metastasis and increases the motility of cancer cells, which makes it a promising marker for diagnosis and prognosis. Furthermore, the overexpression of PFN1 causes breast cancer cells to undergo apoptosis by upregulating PTEN and suppressing AKT activation [49].

The expression of PFN1 plays a crucial role in the progression of breast cancer. When PFN1 levels are reduced, there is a corresponding increase in the motility and invasiveness of breast cancer cells [39]. Interestingly, even a modest increase in PFN1 expression can lead to the formation of actin stress fibers, enhance focal adhesion, and significantly impede the motility and invasiveness of these cells (10.1038/sj.bjc.6604038). In this study, the consistently low levels of PFN1 expression were observed in breast cancer cell, possibly implying that both cell-to-cell and cell-to-matrix adhesion are reduced, leading to increased mobility of breast cancer cells. Furthermore, the progressive downregulation of PFN1 expression with advancing cancer stages supports the existing research that identifies PFN1 as a negative regulator of breast cancer aggressiveness. Conclusively, these findings suggest that PFN1 could be a valuable molecular target for strategies aimed at reducing the aggressiveness of breast cancer. Therefore, we propose further investigation of this tumor-suppressing protein to assess its potential as both a diagnostic and therapeutic target, particularly for Indian patients diagnosed with breast cancer.

Other important DEPs identified in breast cancer serum samples were ANG, PCOLCE, and WFDC3 (upregulated) and FLNA and DSG2 (downregulated). Among upregulated proteins, ANG is a 14-kDa protein, which belongs to the RNase A superfamily. It has been found overexpressed in various cancer types, including breast cancer, and is linked to poor prognosis [50–54]. In particular, patients with invasive urothelial carcinoma who had elevated serum levels of ANG exhibited a significantly lower overall survival rate compared to those with normal ANG levels [50]. PCOLCE, on the other hand, is a secreted glycoprotein, which increases procollagen C-proteinases activity and facilitates the remodeling of the extracellular matrix [55–57]. Higher expression of PCOLCE has been reported across various cancers, and it appears to exert a discernible impact on tumor growth and metastatic progression [55, 58–60]. However, only a few heterogeneous reports exist that establish a correlation between the expression of WFDC3 and tumor progression [61, 62]. Notably, Wu et al. documented the overexpression of WFDC3 gene expression in pancreatic adenocarcinoma, linking it to unfavorable prognosis and diminished infiltration of immune cells [62]. In contrast, Liu et al. identified WFDC3 as a tumor suppressor in colorectal cancer, demonstrating its inhibitory effect on metastasis through an ER*β*-dependent modulation of the TGF*β* signaling pathway [61]. Concerning the downregulated proteins, FLNA is an actin cross-linking protein that exerts inhibitory impact on the progression of breast cancer [63, 64]. A study by Xu et al. elucidated that FLNA regulates the focal adhesion disassembly, consequently curtailing the migration and invasion of breast cancer cells. Moreover, downregulated expression of FLNA was found to be intricately linked with the progression of breast cancer [63]. Another study by Guo et al. reported that FLNA plays a tumor-suppressive role in human breast cancer via the modulation of BRCA1 expression, thereby impeding the progression of malignancy [64]. While the role of FLNA as a tumor suppressor in human breast cancer is evident, a perplexing facet arises from its dual nature in cancer. Its impact on tumor development appears to hinge on its localization within the cell, functioning either as a promoter or a suppressor contingent upon the specific subcellular milieu [65]. Similarly, there are heterogeneous reports about the expression of DSG2 in breast cancer [66, 67]. It is a member of desmogleins, a family of cadherin adhesion and membrane proteins [68]. Davies et al. reported that DSG2 is an important adhesion molecule, and a reduction in its expression increases invasion and motility of breast cancer cells [66]. Conversely, Reimer et al. showed a correlation between DSG2 expression and adverse outcomes, specifically with poor prognosis and an increased risk of recurrence in breast cancer patients [67].

This study identified several DEPs, many of which supported by prior research elucidating similar anomalies, thereby underscoring their significance in the pathogenesis of breast cancer among Indian women. Our focus, however, is on SBSN and PFN1 as potential biomarkers for breast cancer due to their notable sequential changes in expression. Specifically, SBSN consistently showed increased levels in breast cancer samples when compared to controls. Also, its expression increased progressively with the advancement of cancer stages, from EBC to LABC to metastatic stage. In contrast, PFN1 exhibited a decreasing expression in serum samples of breast cancer from EBC to metastatic stage.

## 5. Conclusion

This quantitative proteomics study offers valuable insights into the molecular landscape of breast cancer in Indian women. The identification of dysregulated proteins underscores their significance in the comprehensive understanding of the development of breast cancer. Some of the identified DEPs are also corroborated by available scientific literature, where other investigators have reported similar aberrant expressions of these proteins in breast cancer. Finally, the outcomes of this study propose SBSN and PFN1 as prospective biomarkers for breast cancer among Indian women, warranting further studies to elucidate their precise roles in breast cancer development and the associated molecular mechanisms.

## Figures and Tables

**Table 1:** Patient details.

Features	Particulars	Early breast cancer (EBC)	Locally advanced breast cancer (LABC)	Metastatic breast cancer (M)
Number of participants		5	4	3

Mean age	Years ± SD	57.2 ± 14.09	51.2 ± 5.05	51.3 ± 6.13

Menopausal status	Premenopausal	1 (20%)	1 (25%)	1 (25%)
	Postmenopausal	4 (80%)	3 (75%)	2 (75%)

Side of breast cancer	Right	4 (80%)	0 (0%)	0
	Left	1 (20%)	4 (100%)	3 (100%)

Site of breast cancer	Upper outer quadrant	2 (40%)	0 (0%)	2 (75%)
	Upper inner quadrant	3 (60%)	2 (50%)	1 (25%)
	Lower outer quadrant	0 (0%)	0 (0%)	0 (0%)
	Lower inner quadrant	0 (0%)	0 (0%)	0 (0%)
	Central/Retro-areolar	0 (0%)	2 (50%)	0 (0%)

T stage (T: Tumor size)	Tis	0 (0%)	0 (0%)	3 (100%)
	T1	0 (0%)	0 (0%)	
	T2	5 (100%)	0 (0%)	
	T3	0 (0%)	1 (25%)	
	T4	0 (0%)	2 (50%)	

N stage (N: Number of nearby lymph nodes with cancer)	N0	2 (40%)	0 (0%)	
	N1	3 (60%)	4 (100%)	
	N2	0 (0%)	0 (0%)	1 (25%)
	N3	0 (0%)	0 (0%)	2 (75%)

Type of tumor	Invasive ductal carcinoma	5 (100%)	4 (100%)	2 (75%)
	Invasive lobular carcinoma	0 (0%)	0 (0%)	1 (25%)

Estrogen receptors (ER) status	Positive	3 (60%)	2 (50%)	3 (100%)
	Negative	2 (40%)	2 (50%)	0 (0%)

Progesterone receptors (PR) status	Positive	3 (60%)	2 (50%)	3 (100%)
	Negative	2 (40%)	2 (50%)	0 (0%)

Human epidermal growth factor receptor 2 (HER2) status	Positive	2 (40%)	2 (50%)	1 (25%)
	Equivocal	1 (20%)	0 (0%)	0 (0%)
	Negative	2 (40%)	2 (50%)	2 (75%)

**Table 2:** Differentially expressed proteins in breast cancer serum samples compared to controls as identified by volcano plot analysis using 2 and 0.05 as fold change (FC) and *p* value cut-off, respectively.

Accession	Protein name	FC	log2(FC)	*p* value
*Upregulated in breast cancer*				
Q6UWP8	Suprabasin GN = SBSN	15.743	3.9766	0.001918
P22105	Tenascin-X GN = TNXB	2.1887	1.1301	0.006289
P03950	Angiogenin GN = ANG	2.1019	1.0717	0.030066

*Downregulated in breast cancer*				
P59665	Neutrophil defensin 1 GN = DEFA1	0.49353	−1.0188	0.03702
Q7Z3Y8	Keratin, type I cytoskeletal 27 GN = KRT27	0.48717	−1.0375	0.008975
P80188	Neutrophil gelatinase-associated lipocalin GN = LCN2	0.48343	−1.0486	0.012244
P67936	Tropomyosin alpha-4 chain GN = TPM4	0.47168	−1.0841	0.004098
P29401	Transketolase GN = TKT	0.43887	−1.1881	0.007933
P05164	Myeloperoxidase GN = MPO	0.40197	−1.3148	0.02936
P61204	ADP-ribosylation factor 3 GN = ARF3	0.38389	−1.3812	0.002639
P07737	Profilin-1 GN = PFN1	0.3593	−1.4767	0.000139
P62328	Thymosin beta-4 GN = TMSB4X	0.30531	−1.7117	0.002334
P21333	Filamin-A GN = FLNA	0.28995	−1.7861	0.006752
Q9NQ79	Cartilage acidic protein 1 GN = CRTAC1	0.16475	−2.6017	0.005226
P00558	Phosphoglycerate kinase GN = PGK1	0.10842	−3.2053	7.56E − 05

## Data Availability

The data supporting the findings of this study are available from the corresponding author upon reasonable request.

## Supporting Information

Supporting Information 1 SM 1: Supporting Figures. Figure S1 (a): Principal component analysis (PCA) scores plot considering all proteins, breast cancer vs. controls. (b): Top features identified by partial least squares-discriminant analysis (PLS-DA). (c): Top features identified by orthogonal projections to latent structures discriminant analysis (OPLS-DA). Figure S2 (a): Venn diagram, early breast cancer (EBC) vs controls. (b): Differentially expressed proteins (DEPs) identified by volcano plot. (c): PCA scores plot considering all proteins. (d): PCA scores plot considering only DEPs. (e): Hierarchical clustering analysis (HCA). (f): PLSDA scores plot. (g): Top features identified by PLSDA. (h): OPLS-DA scores plot. (i): Top features identified by OPLS-DA. Figure S3 (a): Venn diagram, prechemotherapy locally advanced breast cancer (LABV1) vs. controls. (b): DEPs identified by volcano plot. (c): PCA scores plot considering all proteins. (d): PCA scores plot considering only DEPs identified by volcano plot analysis. (e): HCA. (f): PLSDA scores plot. (g): Top features identified by PLS-DA. (h): OPLS-DA scores plot. (i): Top features identified by OPLS-DA. Figure S4 (a): Venn diagram, metastatic breast cancer (M) vs. controls. (b): DEPs identified by volcano plot. (c): PCA scores plot considering all proteins. (d): PCA scores plot considering only DEPs. (e): HCA. (f): PLS-DA scores plot. (g): Top features identified by PLS-DA. (h): OPLS-DA scores plot. (i): Top features identified by OPLS-DA. Figure S5: Top features identified by OPLS-DA, EBC vs LABV1. Figure S6 (a): Top features identified by PLS-DA, M vs LABV1. (b): Top features identified by OPLS-DA. Figure S7 (a): Top features identified by PLS-DA, LABV2 vs LABV1. (b): Top features identified by OPLS-DA.

Supporting Information 2 SM 2: Supporting files. Supporting file S1: List of proteins identified by LC-MS/MS analysis. Supporting file S2: Differentially expressed proteins identified by volcano plot analysis. Supporting file S3: Full images for western blots presented in Figure 10.
