# Supplementary material for: Exploring Molecular Alterations in Breast Cancer Among Indian Women Using Label-Free Quantitative Serum Proteomics
Source: Biochem Res Int. 2024 Nov 28;2024:5584607. doi: 10.1155/bri/5584607 (PMC11847613; doi:10.1155/bri/5584607)
Supplement: Supporting Information 1 — SM 1: Supporting Figures. Figure S1 (a): Principal component analysis (PCA) scores plot considering all proteins, breast cancer vs. controls. (b): Top features identified by partial least squares-discriminant analysis (PLS-DA). (c): Top features identified by orthogonal projections to latent structures discriminant analysis (OPLS-DA). Figure S2 (a): Venn diagram, early breast cancer (EBC) vs controls. (b): Differentially expressed proteins (DEPs) identified by volcano plot. (c): PCA scores plot considering all proteins. (d): PCA scores plot considering only DEPs. (e): Hierarchical clustering analysis (HCA). (f): PLSDA scores plot. (g): Top features identified by PLSDA. (h): OPLS-DA scores plot. (i): Top features identified by OPLS-DA. Figure S3 (a): Venn diagram, prechemotherapy locally advanced breast cancer (LABV1) vs. controls. (b): DEPs identified by volcano plot. (c): PCA scores plot considering all proteins. (d): PCA scores plot considering only DEPs identified by volcano plot analysis. (e): HCA. (f): PLSDA scores plot. (g): Top features identified by PLS-DA. (h): OPLS-DA scores plot. (i): Top features identified by OPLS-DA. Figure S4 (a): Venn diagram, metastatic breast cancer (M) vs. controls. (b): DEPs identified by volcano plot. (c): PCA scores plot considering all proteins. (d): PCA scores plot considering only DEPs. (e): HCA. (f): PLS-DA scores plot. (g): Top features identified by PLS-DA. (h): OPLS-DA scores plot. (i): Top features identified by OPLS-DA. Figure S5: Top features identified by OPLS-DA, EBC vs LABV1. Figure S6 (a): Top features identified by PLS-DA, M vs LABV1. (b): Top features identified by OPLS-DA. Figure S7 (a): Top features identified by PLS-DA, LABV2 vs LABV1. (b): Top features identified by OPLS-DA. [file 5584607.f1.pdf]

Exploring molecular alterations in breast cancer among Indian women using label-free quantitative serum proteomics

Anil Kumar Tomar[1], Ayushi Thapliyal[1], Sandeep R Mathur[2], Rajinder Parshad[3], Suhani[3*], Savita Yadav[1*]

Supplementary Figures

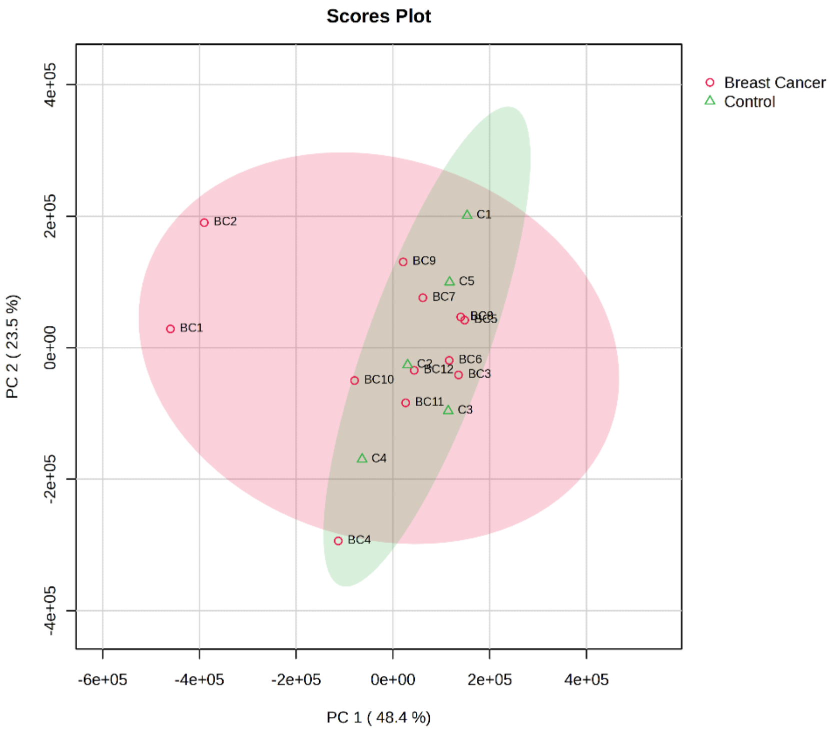

Figure S1 (a): Principal component analysis (PCA) scores plot considering all proteins, breast cancer vs. controls.

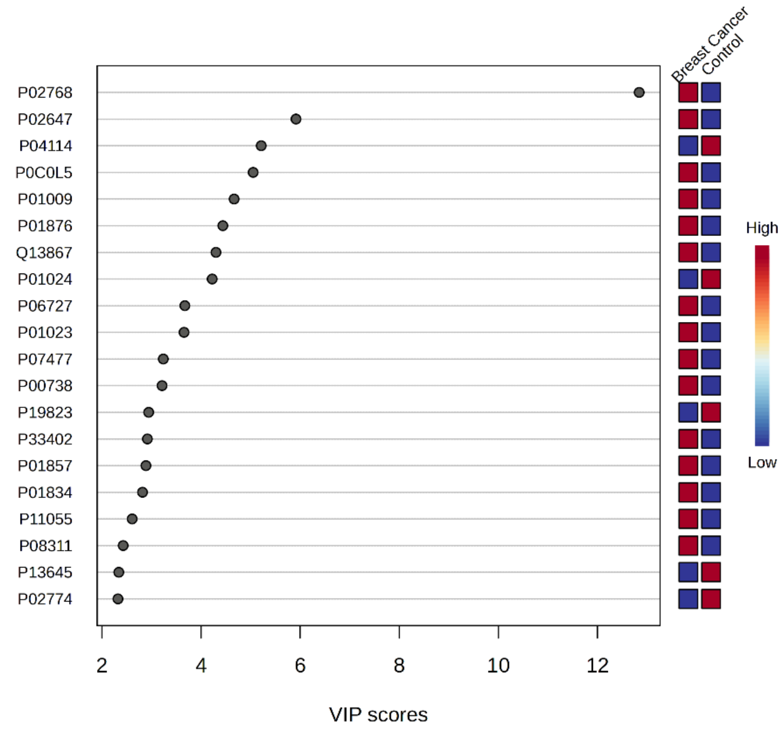

<u>Figure S1 (b):</u> Top features identified by partial least squares-discriminant analysis (PLS-DA), breast cancer vs. controls.

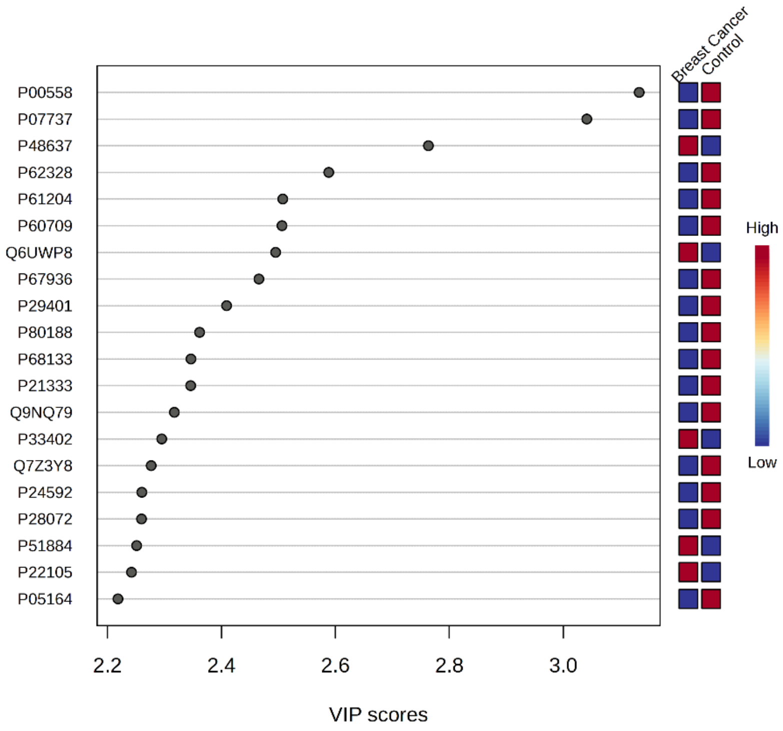

<u>Figure S1 (c):</u> Top features identified by orthogonal projections to latent structures discriminant analysis (OPLS-DA), breast cancer vs. controls.

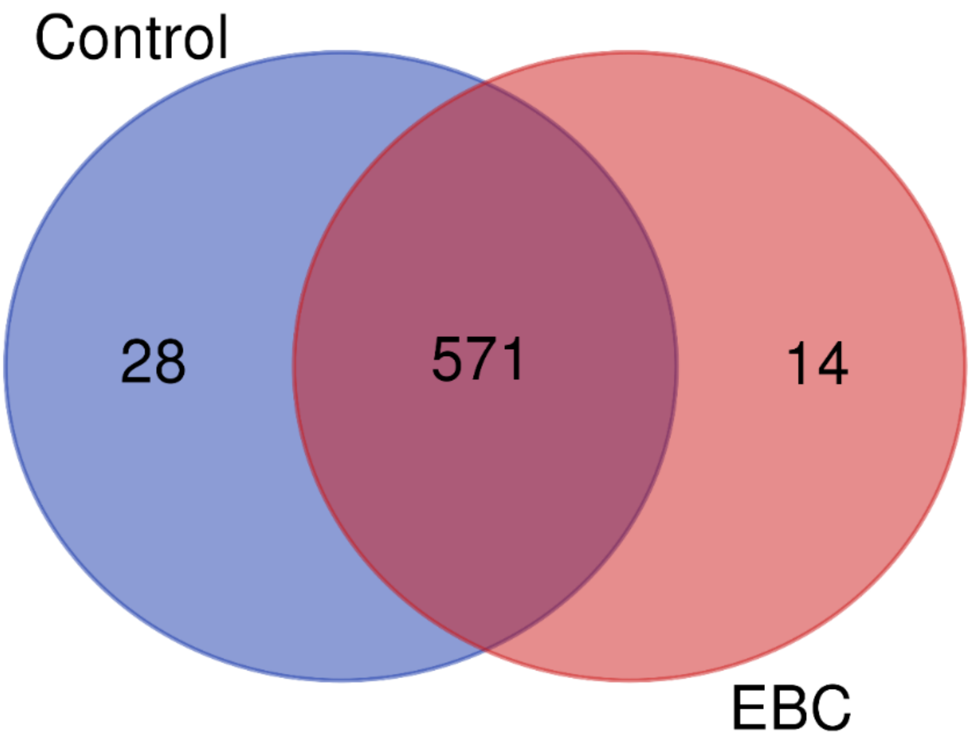

Figure S2 (a): Venn diagram, early breast cancer (EBC) vs controls.

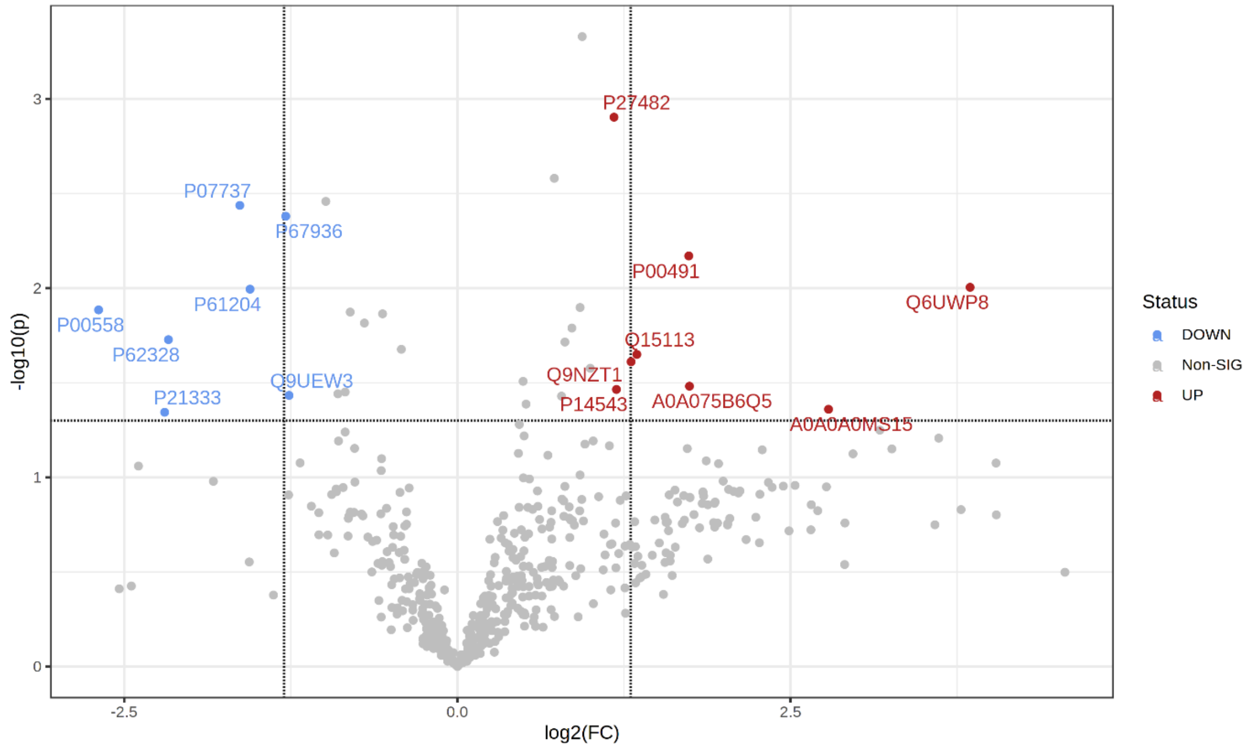

Figure S2 (b): Differentially expressed proteins (DEPs) identified by volcano plot, EBC vs controls.

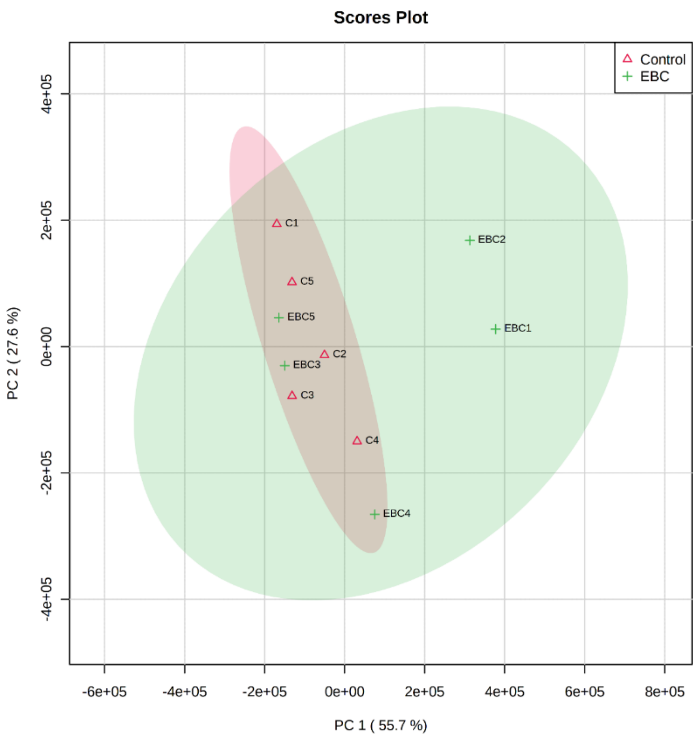

<u>Figure S2 (c):</u> PCA scores plot considering all proteins, EBC vs controls.

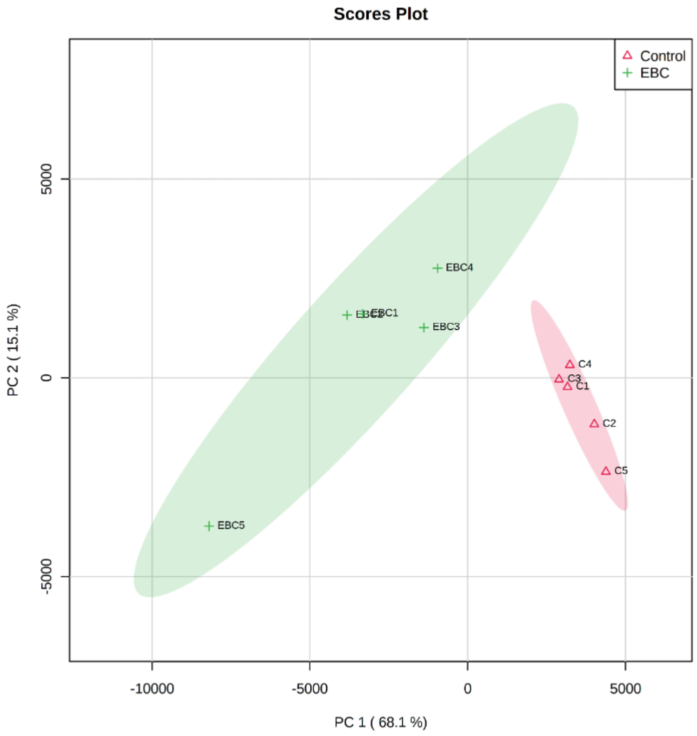

<u>Figure S2 (d):</u> PCA scores plot considering only DEPs, EBC vs controls.

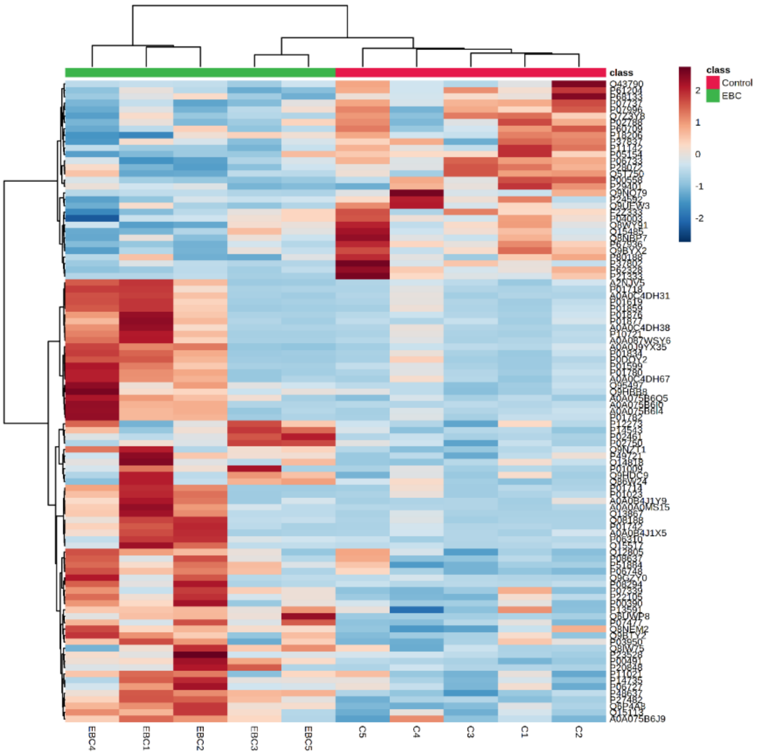

Figure S2 (e): Hierarchical clustering analysis (HCA), EBC vs controls

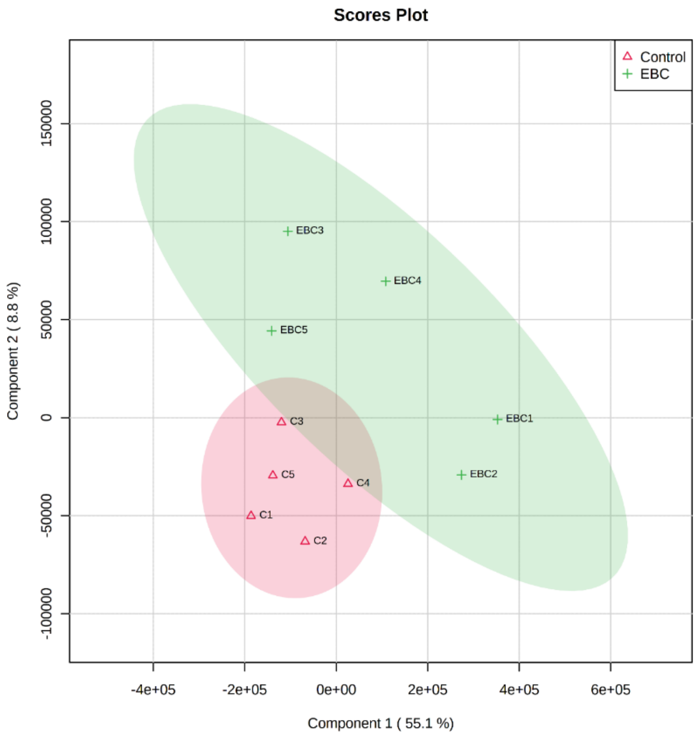

Figure S2 (f): PLSDA scores plot, EBC vs controls.

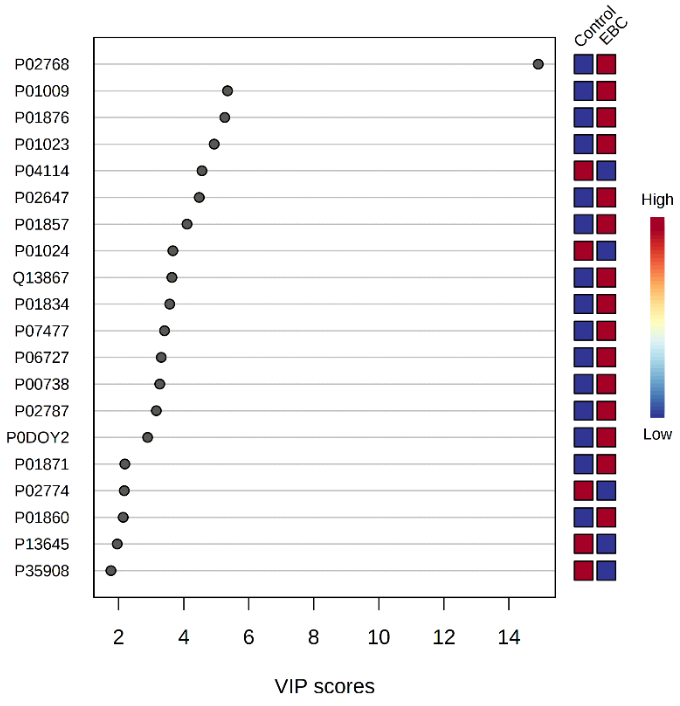

<u>Figure S2 (g):</u> Top features identified by PLSDA, EBC vs controls.

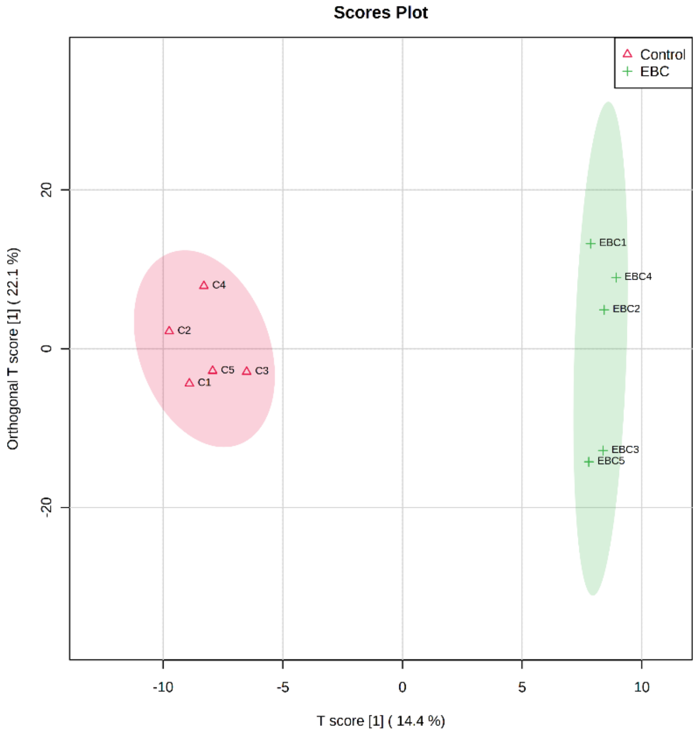

<u>Figure S2 (h):</u> OPLS-DA scores plot, EBC vs controls.

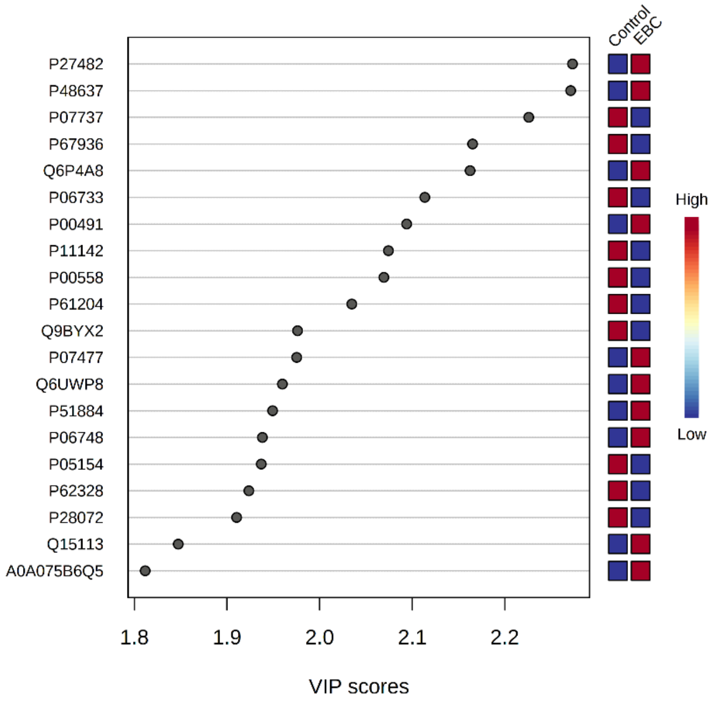

<u>Figure S2 (i):</u> Top features identified by OPLS-DA, EBC vs controls.

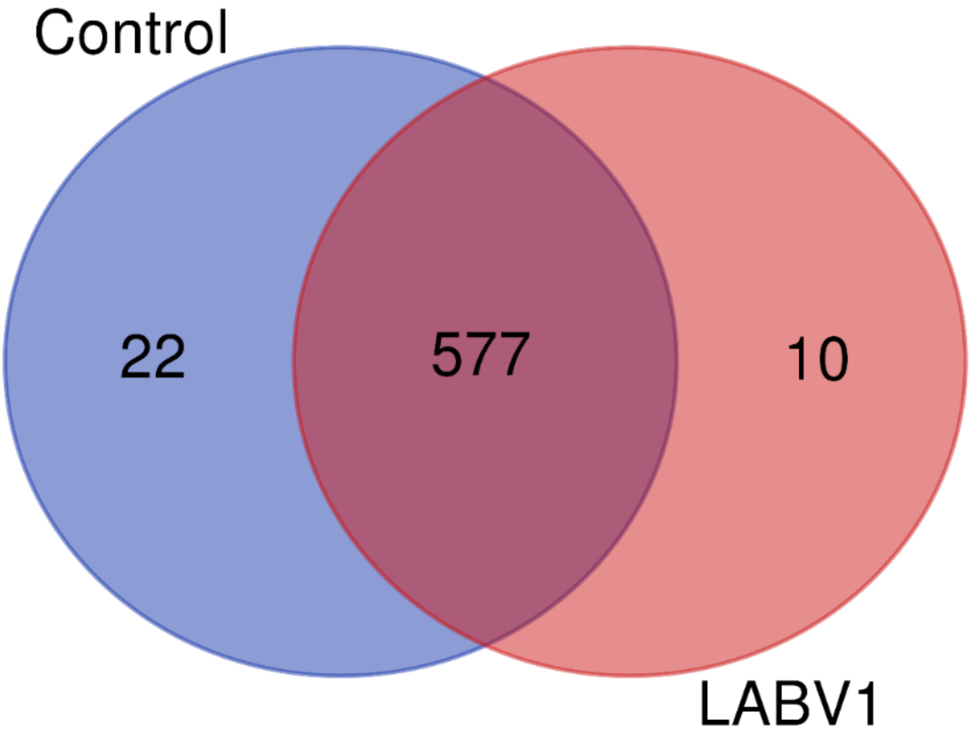

<u>Figure S3 (a):</u> Venn diagram, pre-chemotherapy locally advanced breast cancer (LABV1) vs. controls.

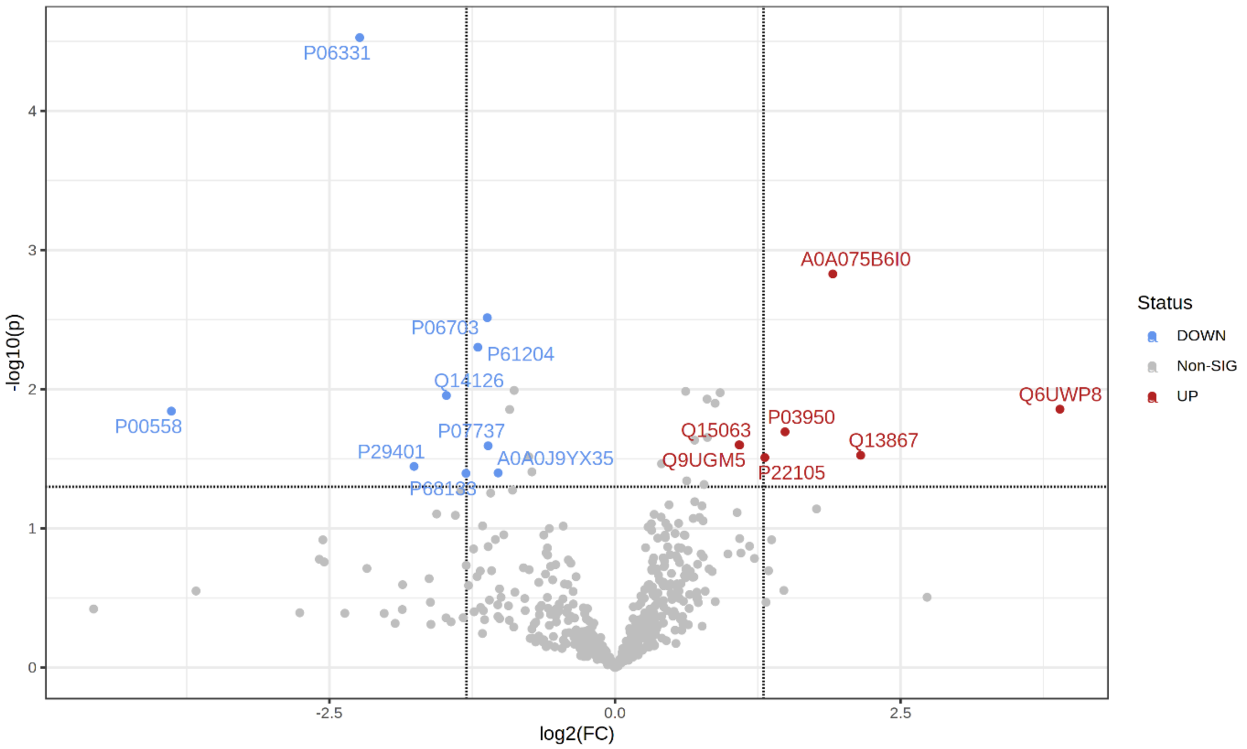

Figure S3 (b): DEPs identified by volcano plot, LABV1 vs controls.

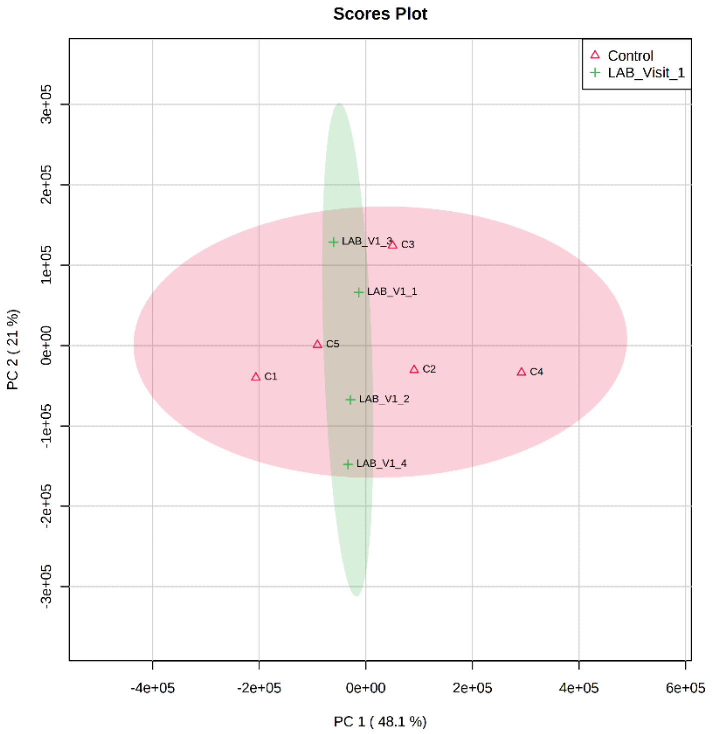

Figure S3 (c): PCA scores plot considering all proteins, LABV1 vs controls

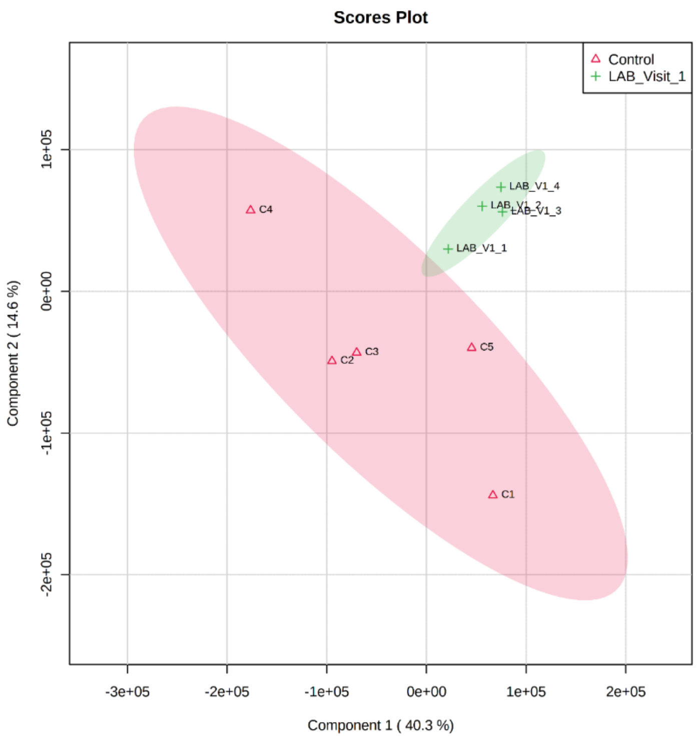

<u>Figure S3 (d):</u> PCA scores plot considering only DEPs identified by volcano plot analysis, LABV1 vs controls

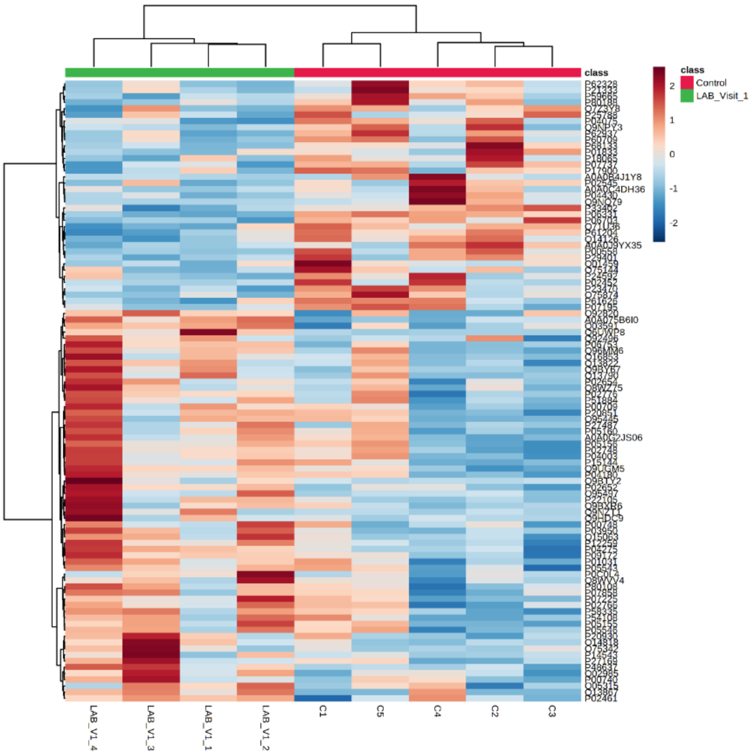

<u>Figure S3 (e):</u> HCA, LABV1 vs controls.

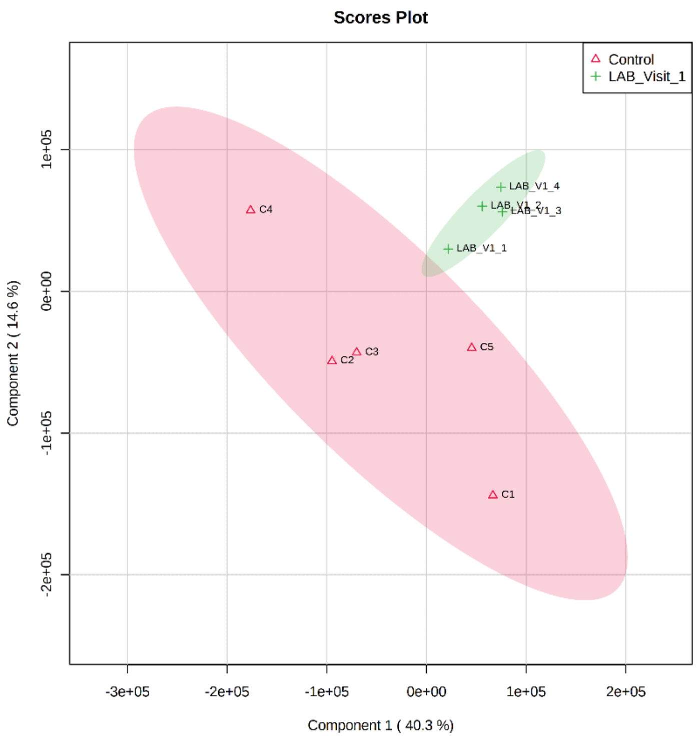

Figure S3 (f): PLSDA scores plot, LABV1 vs controls.

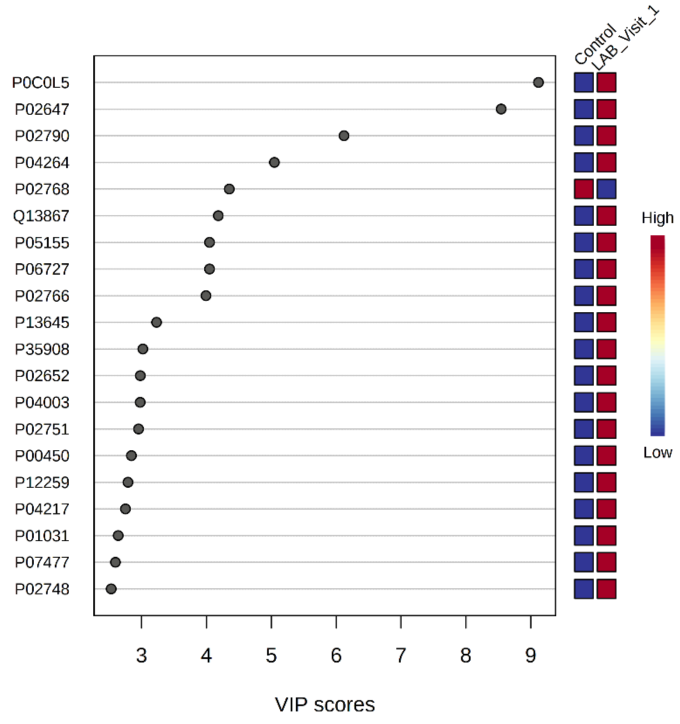

Figure S3 (g): Top features identified by PLS-DA, LABV1 vs controls.

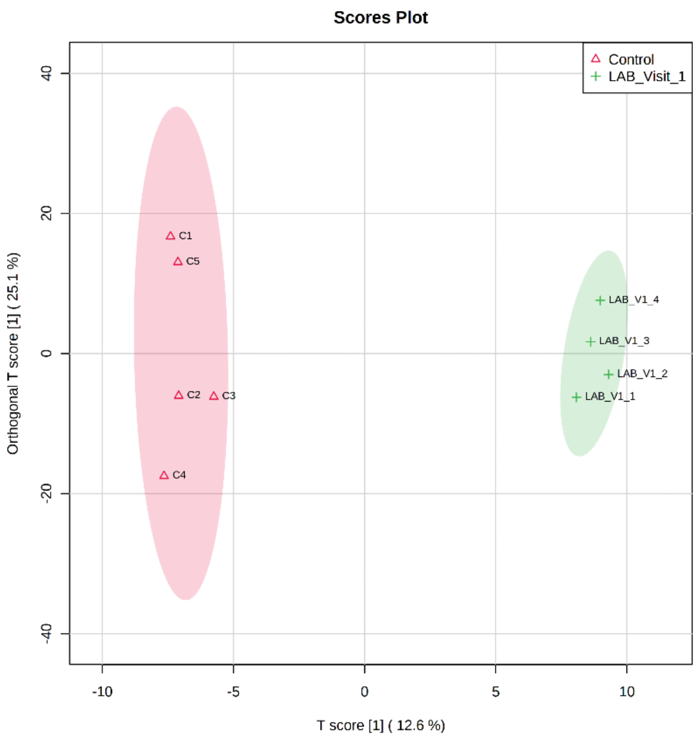

Figure S3 (h): OPLS-DA scores plot, LABV1 vs controls.

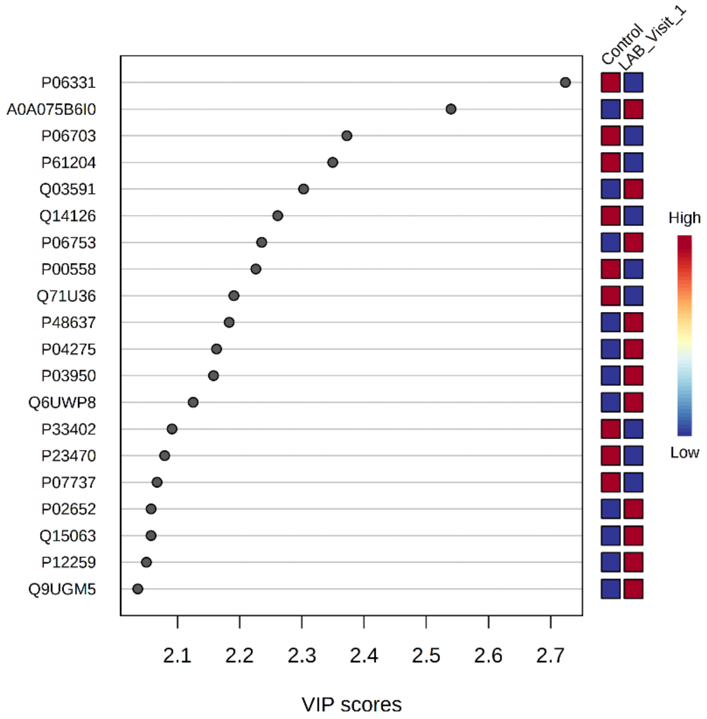

Figure S3 (i): Top features identified by OPLS-DA, LABV1 vs controls

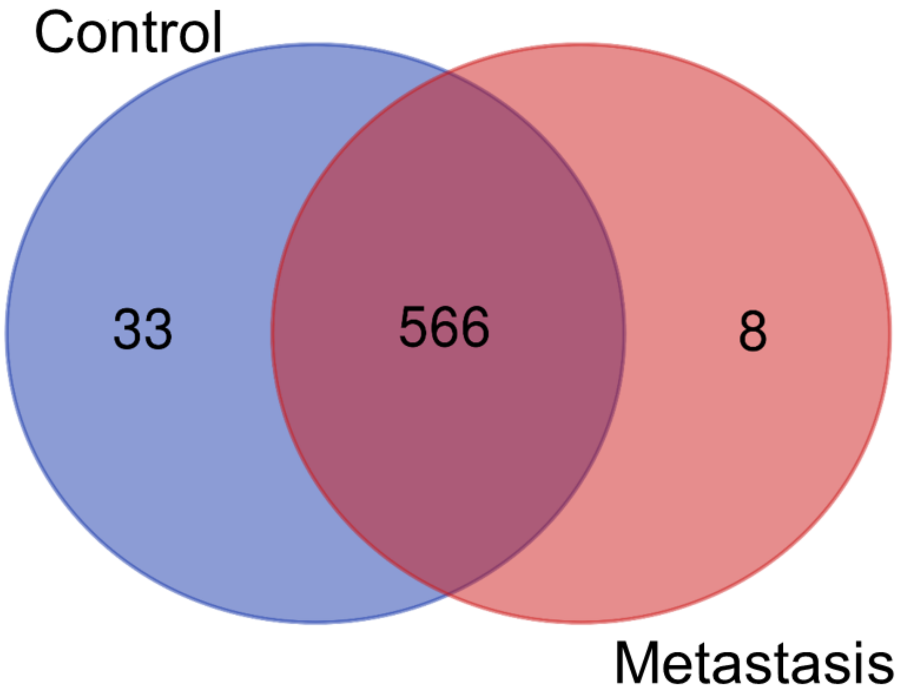

Figure S4 (a): Venn diagram, metastatic breast cancer (M) vs. controls.

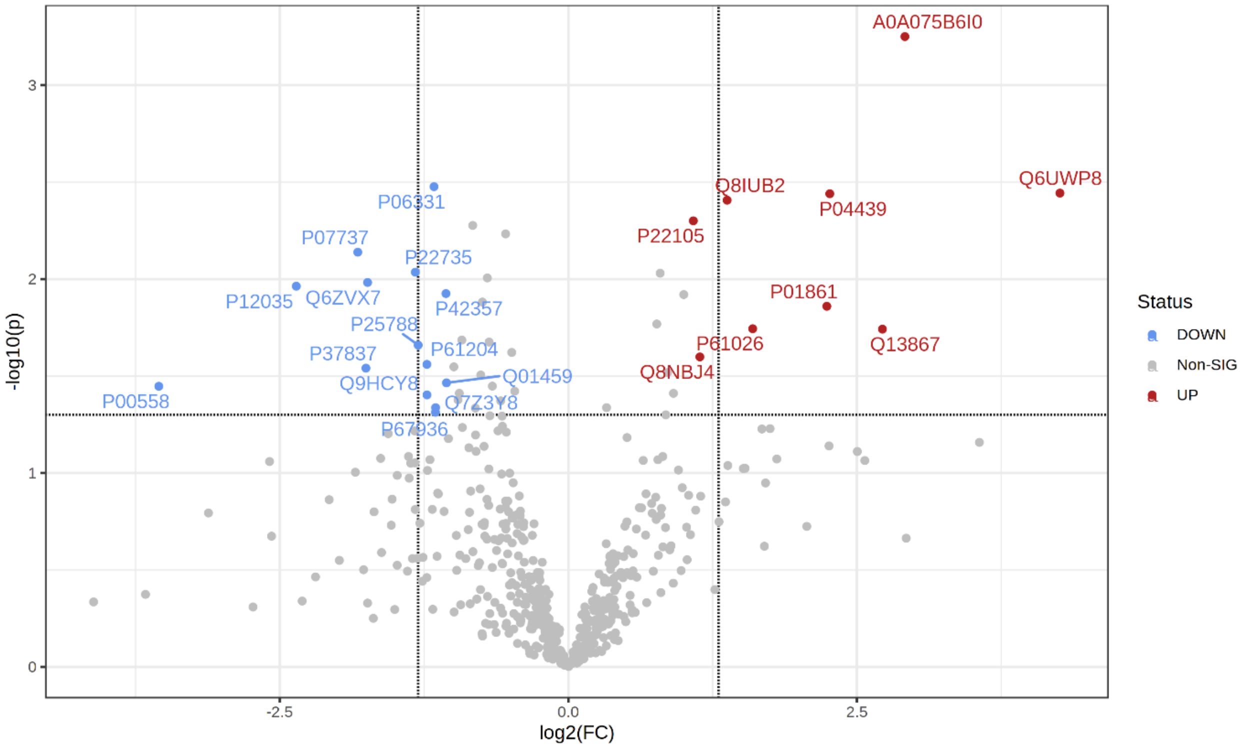

Figure S4 (b): DEPs identified by volcano plot, M vs controls.

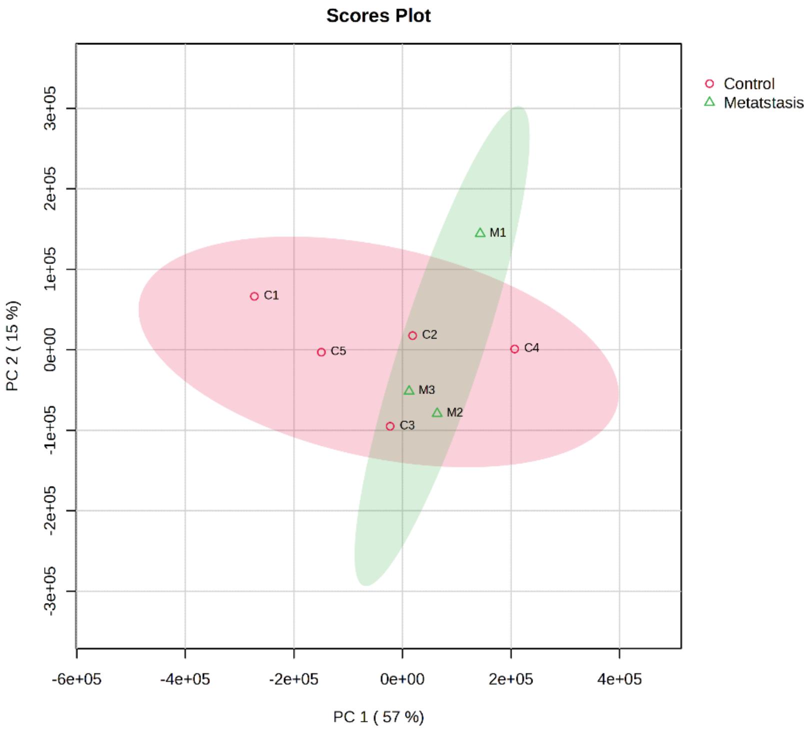

<u>Figure S4 (c):</u> PCA scores plot considering all proteins, M vs controls.

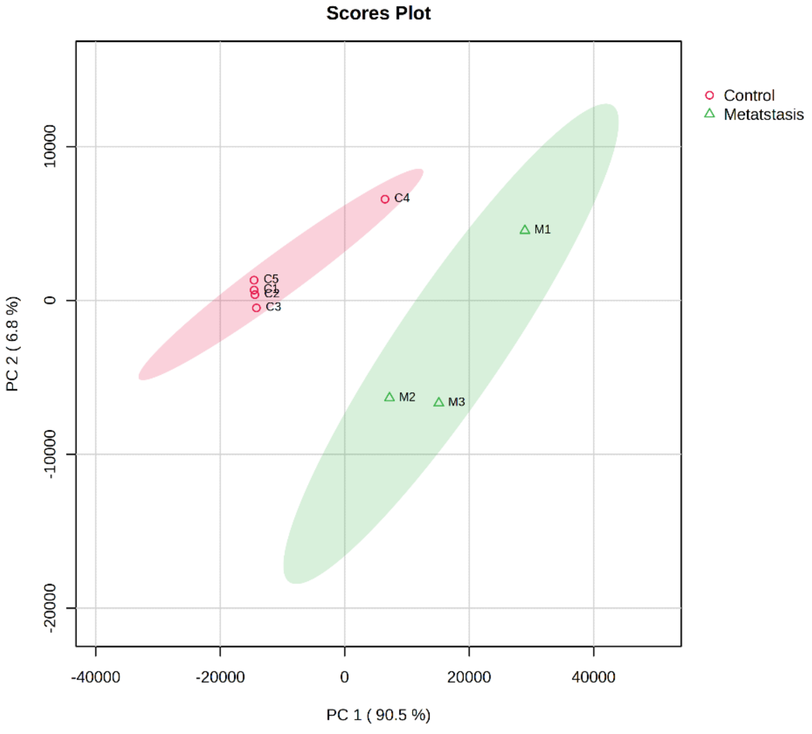

<u>Figure S4 (d):</u> PCA scores plot considering only DEPs, M vs controls.

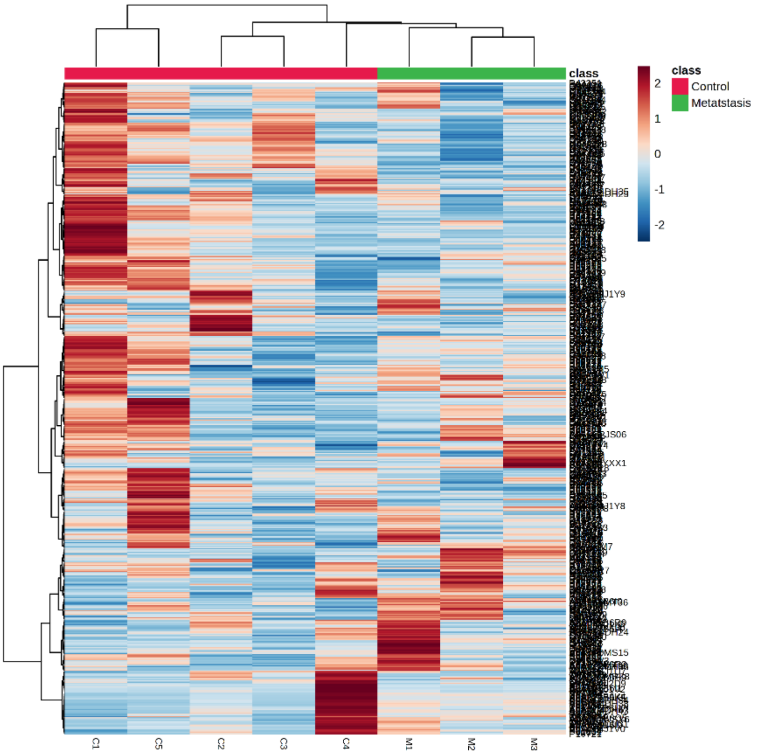

<u>Figure S4 (e):</u> HCA, M vs controls.

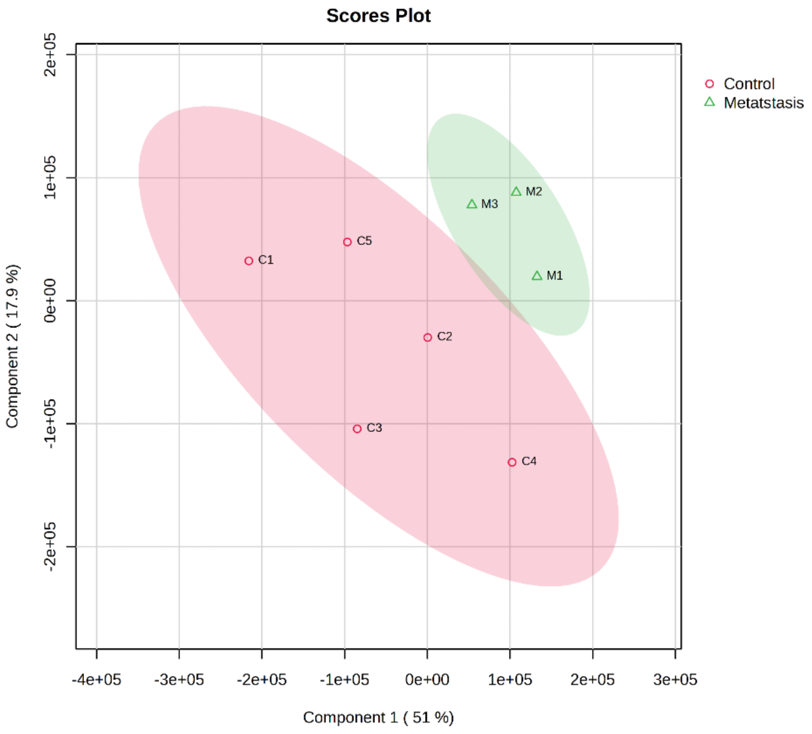

<u>Figure S4 (f):</u> PLS-DA scores plot, M vs controls.

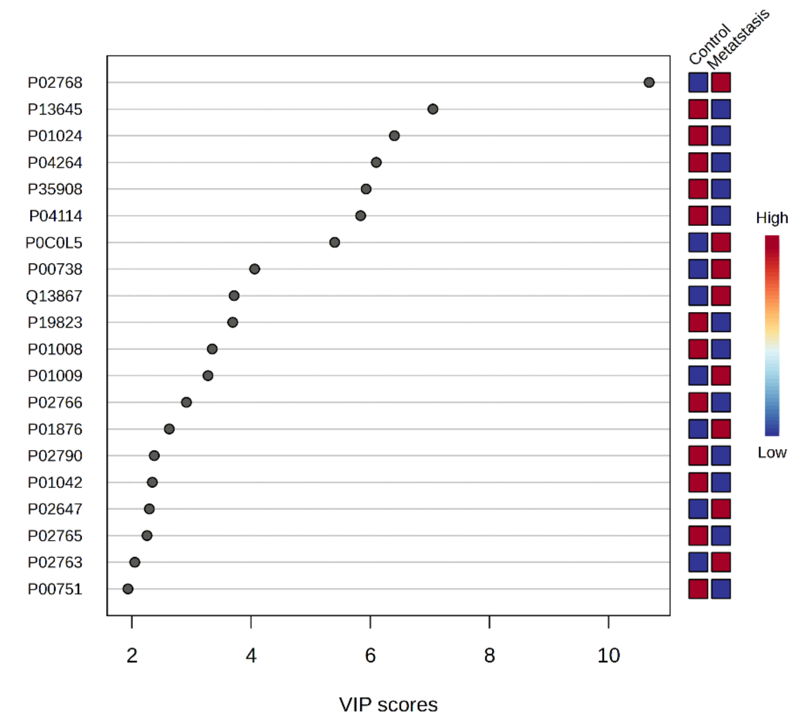

Figure S4 (g): Top features identified by PLS-DA, M vs controls.

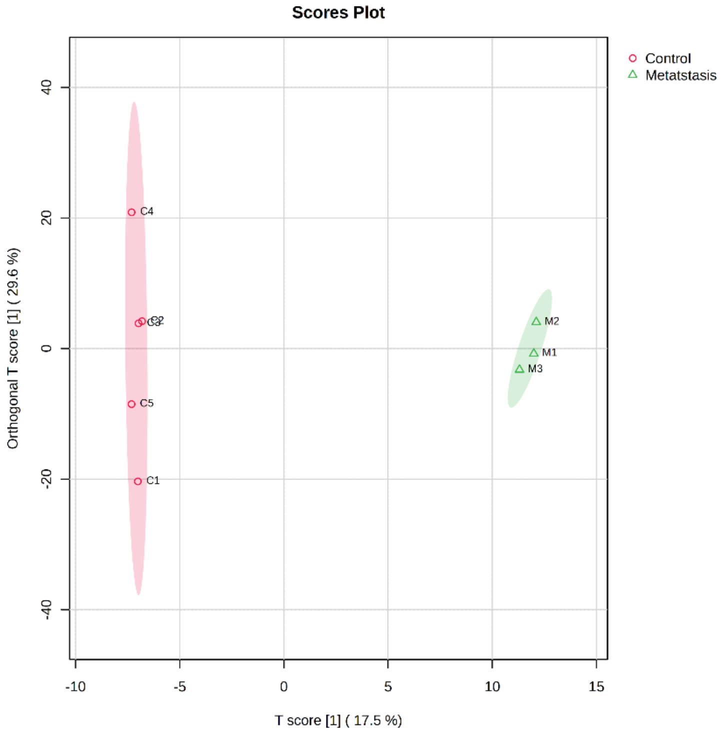

Figure S4 (h): OPLS-DA scores plot, M vs controls.

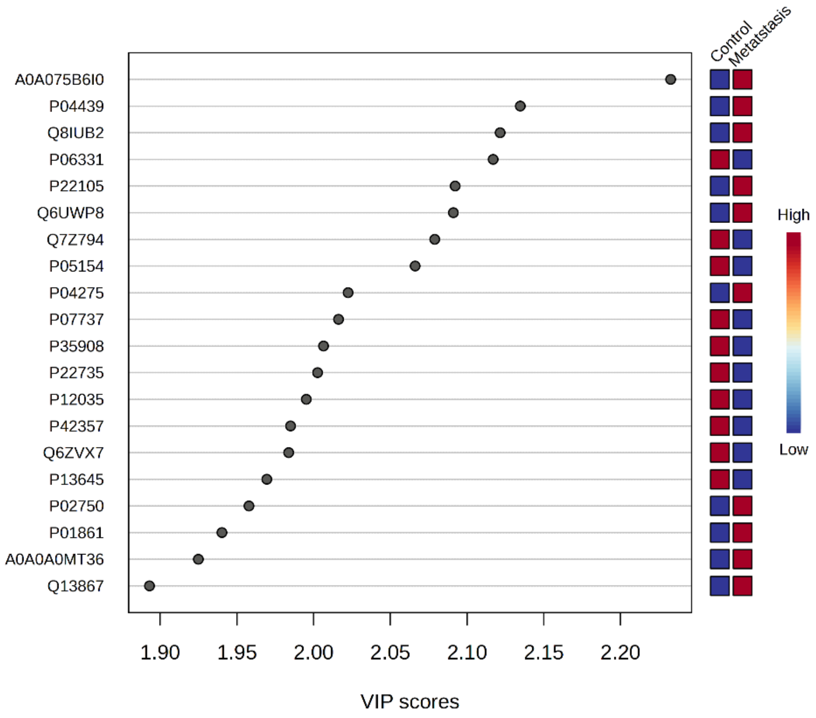

<u>Figure S4 (i):</u> Top features identified by OPLS-DA, M vs controls.

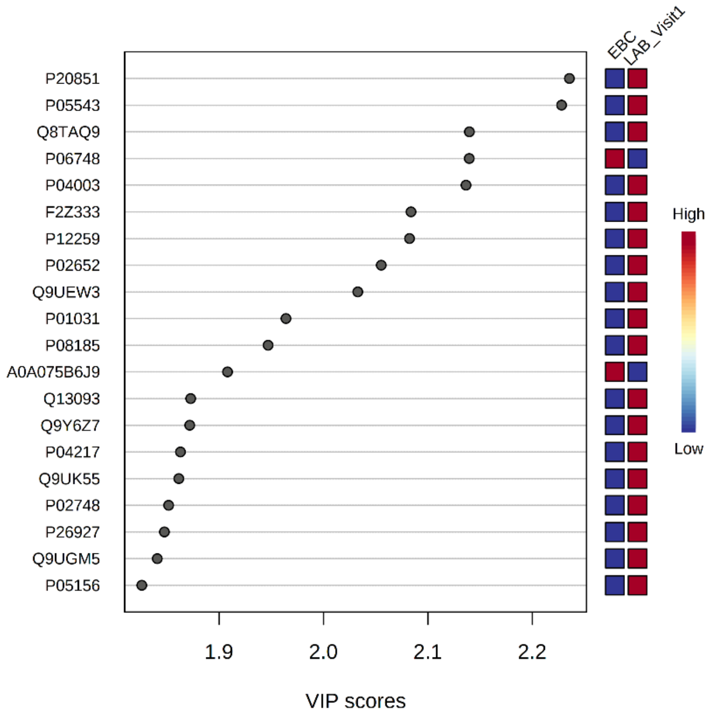

<u>Figure S5:</u> Top features identified by OPLS-DA, EBC vs LABV1.

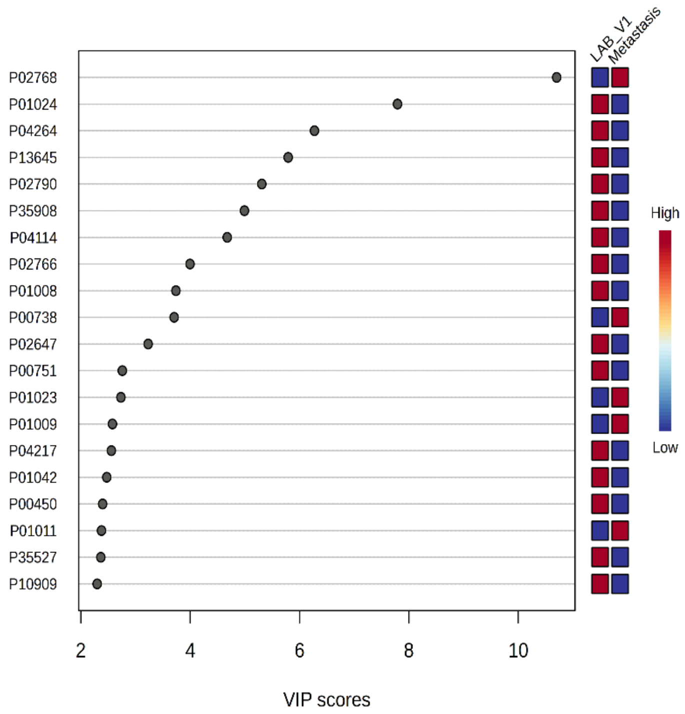

Figure S6 (a): Top features identified by PLS-DA, M vs LABV1.

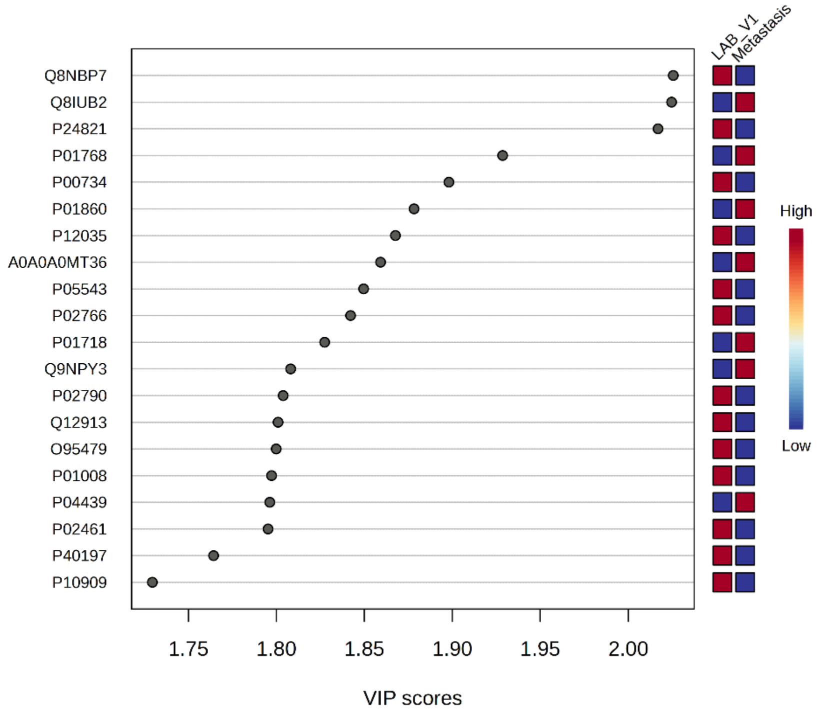

Figure S6 (b): Top features identified by OPLS-DA, M vs LABV1.

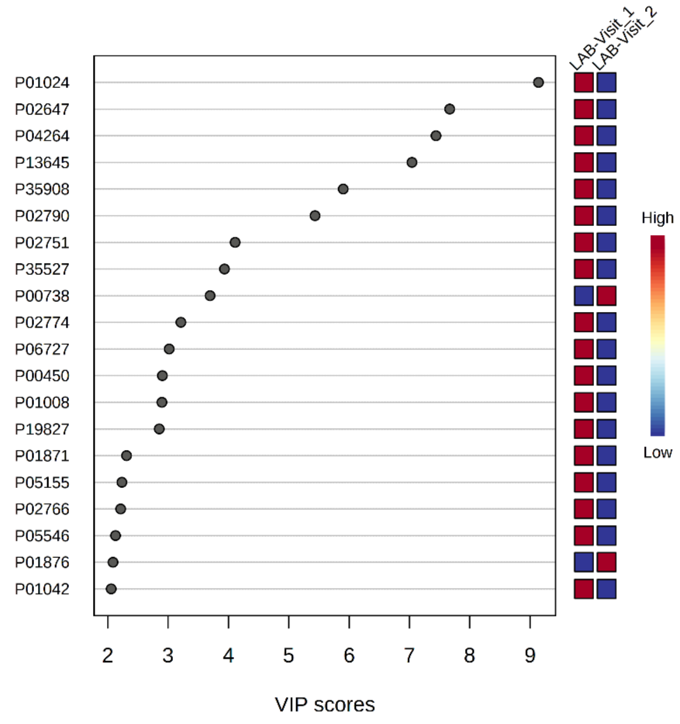

Figure S7 (a): Top features identified by PLS-DA, LABV2 vs LABV1.

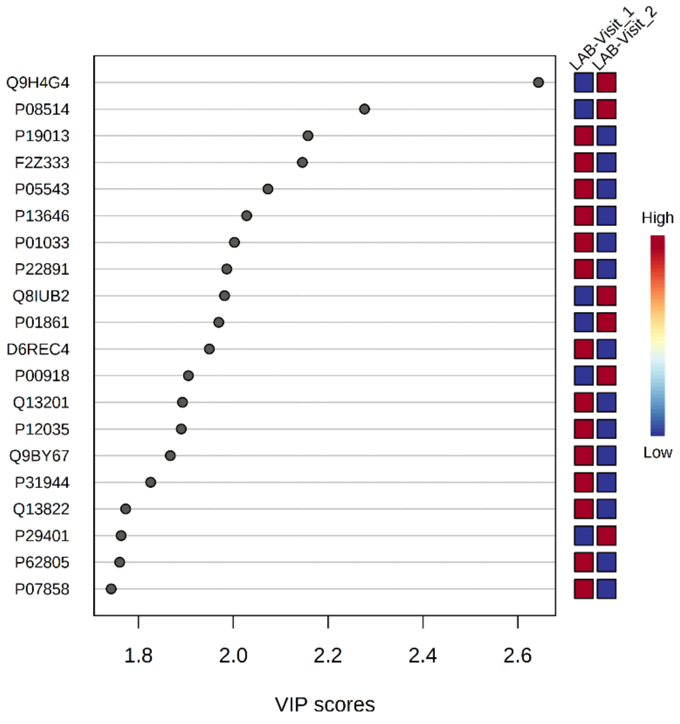

Figure S7 (b): Top features identified by PLS-DA, LABV2 vs LABV1.
