# Supplementary material for: Exploring Molecular Alterations in Breast Cancer Among Indian Women Using Label-Free Quantitative Serum Proteomics
Source: Biochem Res Int. 2024 Nov 28;2024:5584607. doi: 10.1155/bri/5584607 (PMC11847613; doi:10.1155/bri/5584607)
Supplement: Supporting Information 2 — SM 2: Supporting files. Supporting file S1: List of proteins identified by LC-MS/MS analysis. Supporting file S2: Differentially expressed proteins identified by volcano plot analysis. Supporting file S3: Full images for western blots presented in Figure 10. [file 5584607.f2.zip › SM 2_Supplementary file S3.pdf]

Full images for western blots presented in Figure 10

SBSN

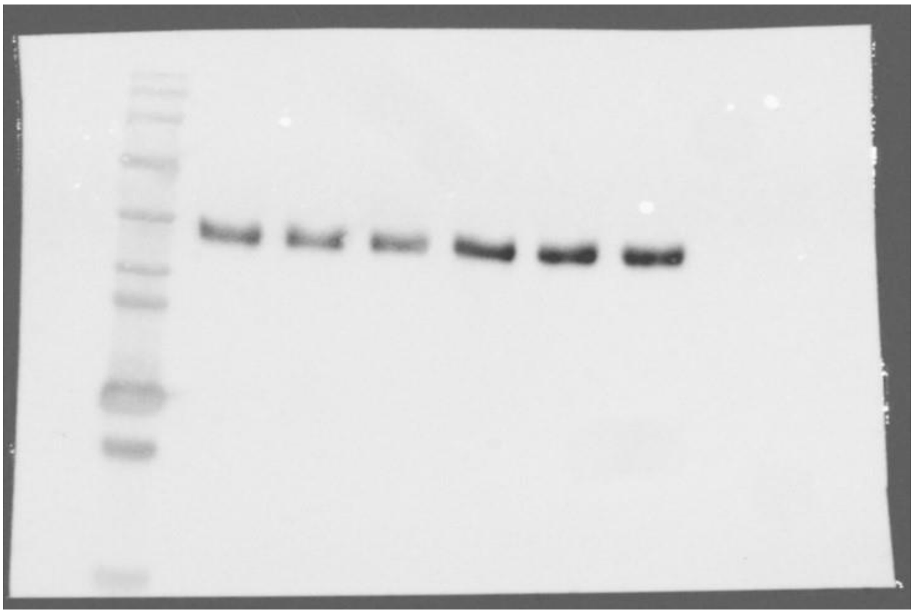

[b] Early Breast Cancer vs Controls

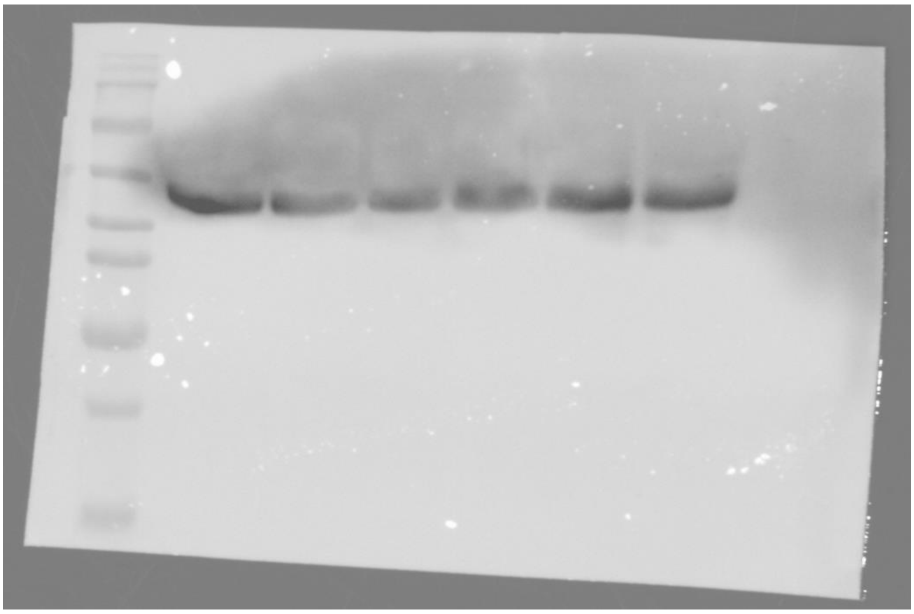

[c] Locally Advanced Breast Cancer vs Early Breast Cancer

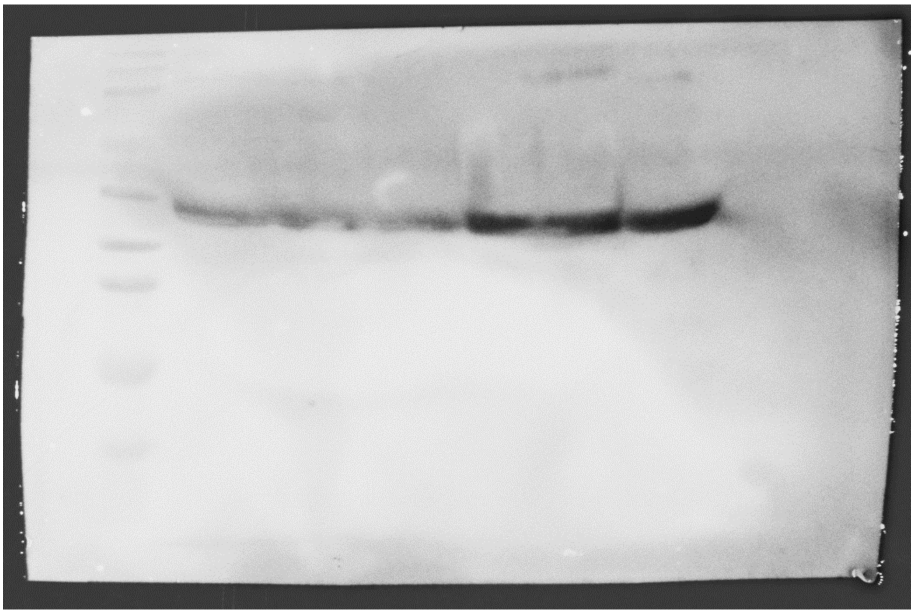

[d] Metastasis vs Locally Advanced Breast Cancer

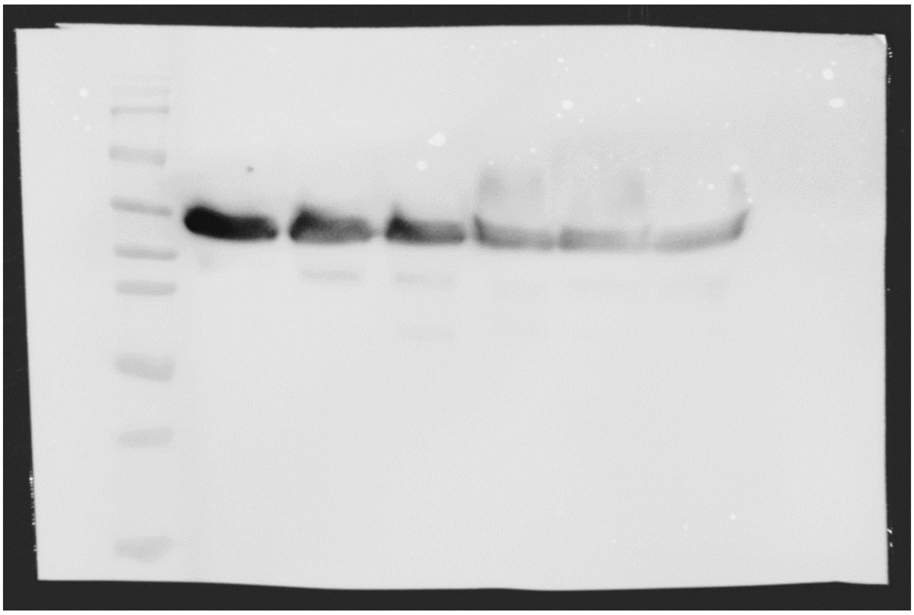

[e] Pre-chemotherapy vs post-chemotherapy locally advanced breast cancer

GAPDH

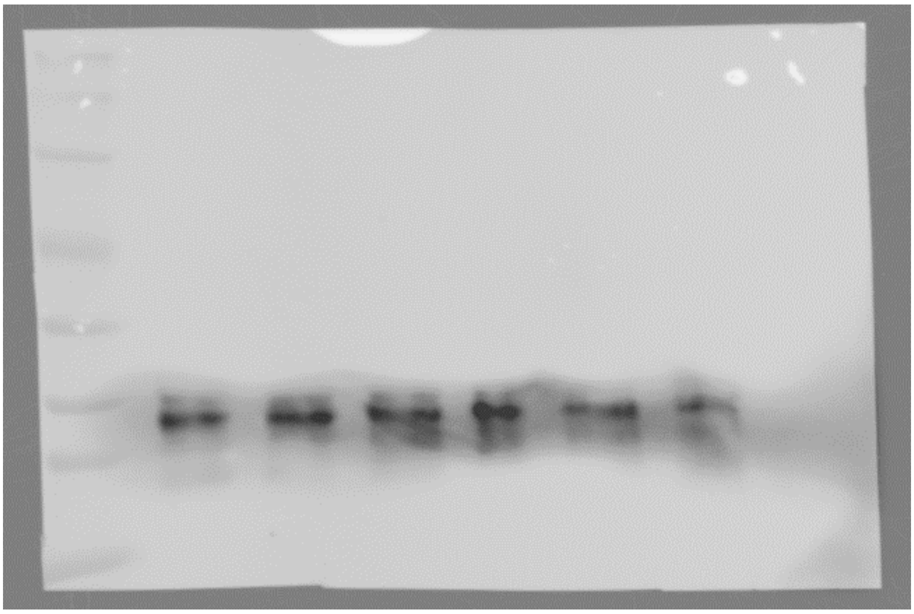

[b]

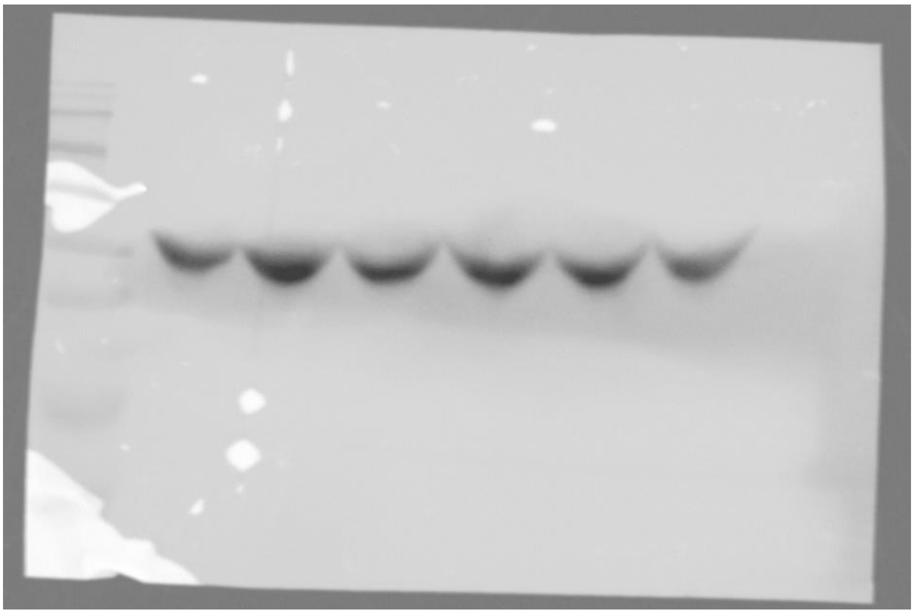

[c]

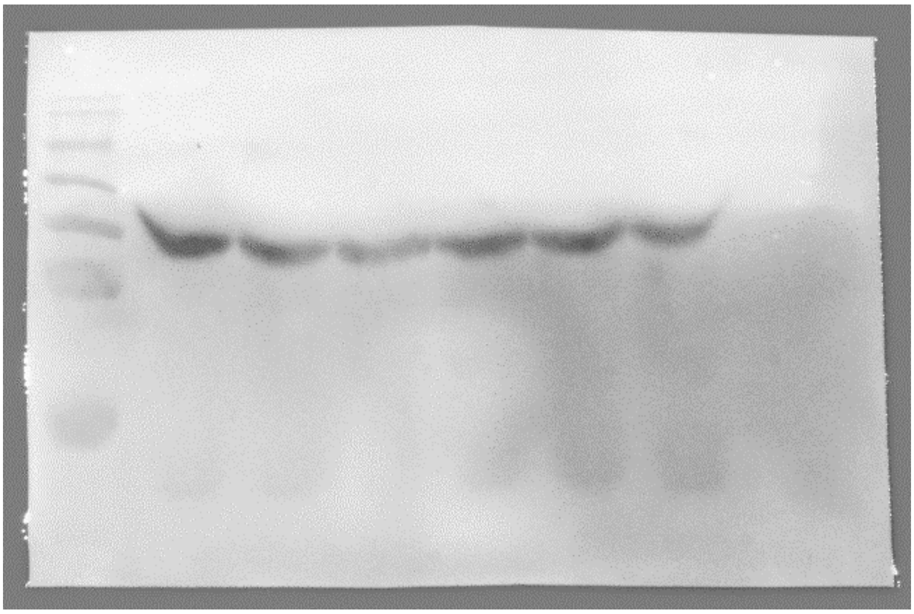

[d]

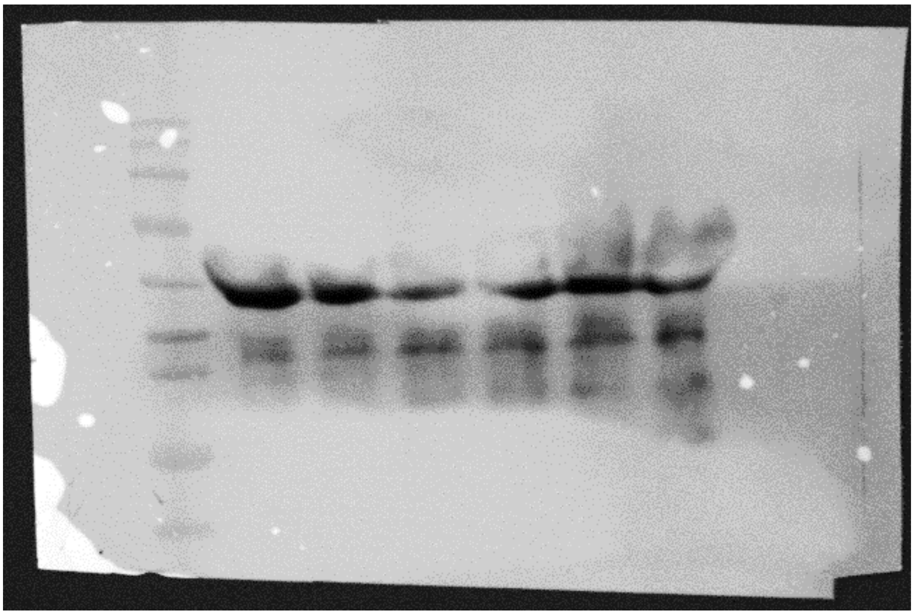

[e]
